# Dissociative Photoionization of 2‑Thiouracil and 4‑Thiouracil: A Molecular Dynamics Study

**DOI:** 10.1021/acs.jpca.5c03342

**Published:** 2025-07-31

**Authors:** Bonasree Roy, Evgenii Titov, Matthew S. Robinson, Markus Gühr, Peter Saalfrank

**Affiliations:** † 426790 University of Potsdam, Institute of Chemistry, Karl-Liebknecht-Straße 24-25, Potsdam 14476, Germany; ‡ 339694 European XFEL, Holzkoppel 4, Schenefeld 22869, Germany; § 28332 Deutsches Elektronen-Synchrotron (DESY), Notkestraße 85, Hamburg 22607, Germany; ∥ Institut für Physikalische Chemie, Universität Hamburg, Grindelallee 117, Hamburg 20146, Germany

## Abstract

Thionated nucleobases have drawn considerable attention due to their role in medical treatment and biology, which triggered studies of their photophysics and photochemistry using various experimental and theoretical techniques. In particular, vacuum ultraviolet (VUV)-induced dissociative photoionization of 2-thiouracil (2-TU) has been recently studied using synchrotron radiation [Robinson et al., *Molecules* **2023**, 28, 2354]. Here, using molecular dynamics simulations at the semiempirical OM2 level, we study the fragmentation dynamics of the cations of 2-TU and its isomer, 4-thiouracil (4-TU). Considering various VUV photon energies, we calculate energy-resolved mass spectra and breakdown diagrams for 2-TU and 4-TU. Remarkably, we find that the major fragments are different between the two compounds: 69 amu (C_3_NH_3_O^+^) for 2-TU and 85 amu (C_3_NH_3_S^+^) for 4-TU. Our simulations provide direct mechanistic insight into this observation and the fragmentation processes of thionated uracils in general.

## Graphical abstract

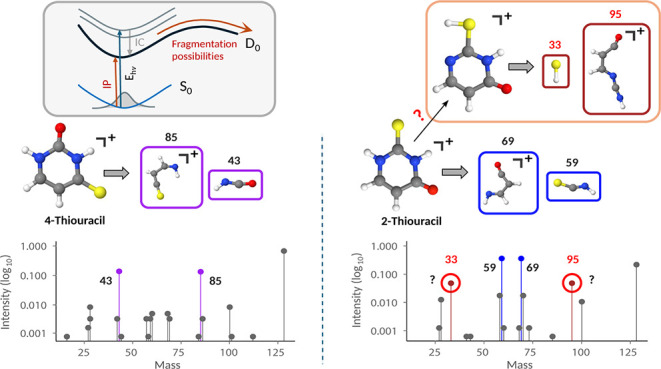

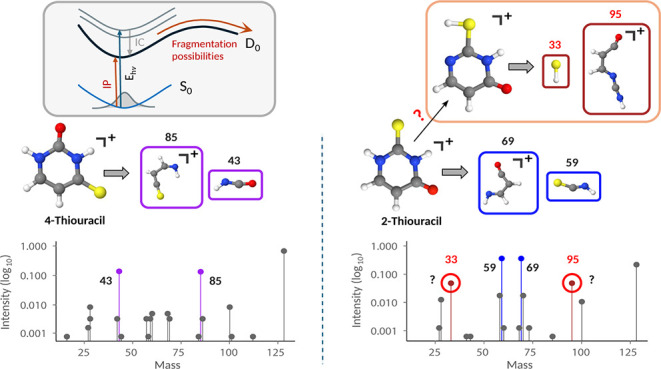

## 1 Introduction

Thiouracil and its derivatives have attracted considerable attention due to their significant biological and chemical roles. As sulfur-containing compounds, classified as thionucleobases, thiouracils are essential in the synthesis of various pharmaceutical agents^1–3^ and have been extensively studied for their distinctive photochemical properties.^4–7^ Thionated nucleobases (natural or synthetic) are candidates for radiation and photodynamic therapies.^8–10^

This practical relevance has driven extensive experimental and theoretical research aimed at understanding their fundamental photochemical and photophysical characteristics. Among these, 2-thiouracil has been a focal point for numerous studies due to its intriguing properties and potential applications.^4,11,12^ The unique photophysical behavior of thionucleobases, including a redshift in UVA absorption, is associated with the substitution of oxygen by sulfur.^4,10,13,14^ Sulfur is also the cause of a fast relaxation decay to the triplet state via intersystem crossing in photoexcited 2-thiouracil.^15,16^ Research has focused on elucidating their photodynamics by comparing the excited-state potential energy surfaces and relaxation pathways of thionated nucleobases with those of native nucleobases.^17–25^

Another structurally similar thionucleobase to 2-thiouracil is 4-thiouracil, which has received relatively less attention in the scientific community. 4-Thiouracil stands out among thionucleobases for its cytostatic properties and versatile applications as a biological photoprobe and regulator of transcription.^26–28^ Its ability to undergo photo cross-linking reactions with nucleic acids makes it particularly valuable as a site-specific optical probe in cross-linking studies, thanks to its strong absorption of UVA light around 330 nm.^4,13^ Also, theoretical simulations of its nonadiabatic dynamics and X-ray signals have been recently reported.^29,30^

In this work, we study the dynamics of 2-TU and 4-TU after single-photon VUV-induced photoionization. Our emphasis is on a comparative study of the two isomers and on bond-breaking processes in general, also related to mass spectrometry. Our molecular dynamics investigation focuses on fragmentation patterns and mechanisms and how different vacuum ultraviolet (VUV) photon energies affect the underlying processes and channels. For 2-TU, we also compare our computational results with the recently reported experiments by Robinson et al.^24^

Our paper is organized as follows. In the next section, Section 2, computational methods to study the photofragmentation of the mentioned cations are described, based on (adiabatic or nonadiabatic) molecular dynamics in conjunction with semiempirical electronic structure theory. In Section 3, we present results from adiabatic molecular dynamics calculations, for 2-TU (Section 3.1) and 4-TU cation’s photofragmentation (Section 3.2), including fragmentation patterns, breakdown diagrams, and reaction mechanisms. Further discussion is provided in Section 3.3. A comparison to the experiment is made in Section 3.4. Possible nonadiabatic effects are discussed in Section 3.5. Finally, Section 4 concludes this work.

## 2 Computational Details

Computationally, we start from the geometries of 2-TU and 4-TU in their neutral ground states (S_0_), which have been optimized with the B3LYP hybrid functional^31,32^ of density functional theory (DFT), using a 6-31G* ^33–35^ basis set, as shown in Figure 1. The Gaussian 16 program^36^ was used for these calculations.

**Figure 1:**
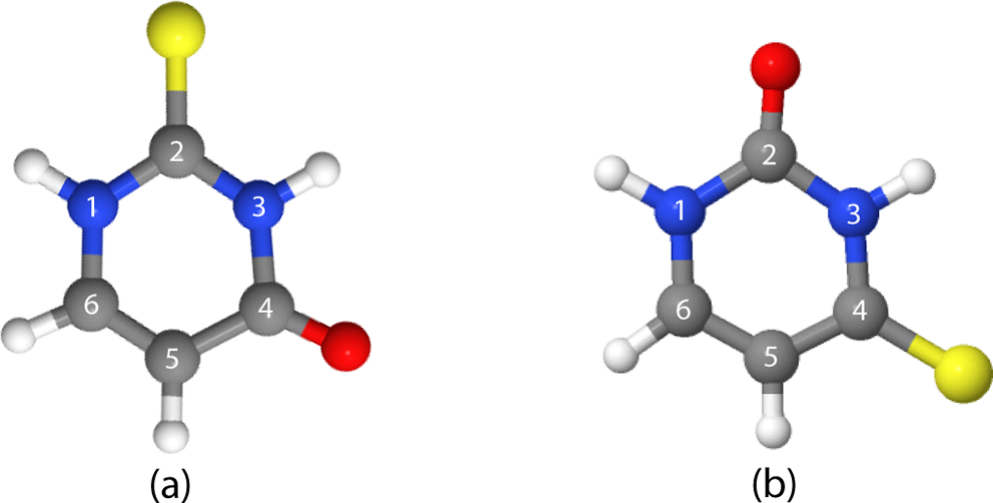
Optimized geometries of neutral (a) 2-thiouracil and (b) 4-thiouracil at the B3LYP/6-31G* level of theory.

The molecular dynamics (MD) before and after photoionization were modeled using the semiempirical OM2 ^37^ method implemented in the MNDO program,^38^ which was combined with our in-house code for trajectory propagation.^39^ A semiempirical quantum chemical method was used in view of the large number of trajectories needed to be calculated and time scales to be explored (see below). The OM2 method was selected because of its good performance for ionization potentials (IP) when compared to the experimental results, e.g., 8.73 eV for 2-TU.^24^ (We note, though, that, in principle, the agreement with a reference result may be achieved by reparameterization of a semiempirical method, which we did not perform in this work.)

We follow two approaches for the post-ionization dynamics: In the first, we solve the classical equations of motion for all atoms in the (suddenly created) cationic ground state, D_0_, with excess energy provided by the (hypothetical) exciting VUV photon being converted to nuclear kinetic energy. This first approach is similar in spirit to Grimme’s Quantum Chemistry Electron Ionization Mass Spectra (QCEIMS) method for computing electron ionization mass spectra.^40^ The second approach goes beyond the first one and also allows for the excitation of higher-energy cationic states D_*i*_ (*i* > 0) and subsequent nonadiabatic coupling to other cationic states including D_0_. This will be taken into account by a nonadiabatic surface hopping (NASH) approach. If the internal conversion from higher doublet states to the doublet ground state of the cation is fast, then fragmentation processes will take place in D_0_ mostly, making the first approach, which is computationally much less demanding, sufficient. This assumption is often invoked when studing dissociative photoionization.^41^

Specifically, the workflow for the first adiabatic approach is as follows:

(1) Run ground-state (S_0_) Born–Oppenheimer MD (BOMD) at constant temperature of 450 K (the temperature used in the experiment^24^) for the neutral molecule for 100 ps and sample geometries for subsequent photofragmentation dynamics. The S_0_ dynamics were evolved on the OM2 S_0_ potential energy surface (PES), starting from the B3LYP/6-31G* optimized geometries. (We note that the choice between B3LYP or OM2 optimized geometries has negligible impact on the BOMD outcomes, as the system thermalizes rapidly during the simulation.) The S_0_ energies were calculated using spin-restricted OM2 (ROM2). The temperature was controlled by a simple velocity-rescaling algorithm.^39^
(2) Set the photon energy, *E*_*hν*_. VUV energies in the range 12–16 eV have been considered based on the experimental study by Robinson et al.^24^
(3) Calculate the vertical ionization potential, IP, as the energy difference between the ground state of the cation, D_0_, and the neutral ground state, S_0_, for each of the initial geometries. The D_0_ energy was calculated using spin-unrestricted OM2 (UOM2).
(4) Calculate the difference *E_hν_* – IP for each of the initial geometries. This difference is then assumed to go completely into nuclear kinetic energy *E*_kin_, assuming that nonadiabatic effects can be neglected and/or fast internal conversion to D_0_ already took place.
(5) Sample random velocities corresponding to the nuclear kinetic energy *E*_kin_ calculated in (4) for each initial geometry. We note that by doing so, we do not account for the initial nuclear kinetic energy of the neutral $E_{\mathrm{kin}}^{\mathrm{ini}}$ . At *T* = 450 K, this energy is ∼0.58 eV (calculated as $E_{\mathrm{kin}}^{\mathrm{ini}}=\frac{3N-6}{2}kT$ , where *N* is the number of atoms, *N* = 12 in our case). Therefore, the photon energies in (2) should be shifted down by 0.58 eV when comparing with the experiment, i.e., 11.42–15.42 eV instead of 12–16 eV. In what follows, we use the latter (12–16 eV) for simplicity.
(6) Propagate many trajectories (1000 for a given *E*_*hν*_) for the cation (in the D_0_ state) at constant energy for 10 ps [(much) longer time scales are computationally prohibitive]. The velocity Verlet algorithm was used to integrate trajectories with a time step of 0.5 fs. The D_0_ BOMD were propagated at the UOM2+Fermi smearing level, since Fermi smearing^42^ was found to significantly remedy energy conservation problems observed for pure UOM2.^39^ Despite this improvement, some deviations in energy (violation of energy conservation) may still occur. Therefore, we further used an additional criterion for total energy deviation to select “good” trajectories for subsequent analysis: $\underset{t}{\max}\left[E\left(t\right)-E\left(0\right)\right]\leq 0.1\mathrm{eV}$ , where *t* is time and *E* is the total energy.
(7) Analyze the trajectories: count all fragments (ions and neutrals) and calculate the relative abundance; inspect fragments and reaction mechanisms. The distance between atoms used to judge on fragmentation was set to 4 Å. The relative abundance of fragment *i* is calculated as
1 $$F_{i}=\frac{N_{i}}{\underset{j}{\sum }N_{j}}$$
  where *N*_*i*_ is the number of times fragment *i* was detected in all trajectories, and the sum in the denominator goes over all observed fragments.
(8) To compute mass spectra, identify cationic fragments using Stevenson’s rule.^43,44^ Accordingly, a positive charge +1e is assigned to the fragment with the smallest IP. Calculate energy-resolved mass spectra, and construct breakdown diagrams. A signal at particular m/z in the mass spectrum is calculated using eq 1 but with *i* labeling cationic fragments only (*z* = 1 is always assumed and the sum in the denominator goes over the cations only and thus equals the total number of trajectories).

To obtain preliminary insight into nonadiabatic effects and relaxation, we performed as a second approach surface hopping^45^ simulations for several trajectories at the restricted open-shell OM2 configuration interaction singles and doubles level of theory, denoted as ROOM2/CISD(5 × 5), where 5 × 5 represents the active space considered, having 5 highest occupied and 5 lowest unoccupied orbitals (the orbitals are shown in Figure S9). The surface hopping simulations were done with the MNDO program^46^ where 11 electronic states D_0_–D_10_ were included in the simulations. Several different excited cationic states were considered as the initial states, as detailed in Section 3.5. The energy-based decoherence correction^47^ was used. The trajectories were propagated for 1 ps with a nuclear time step of 0.1 fs and an electronic time step of 1 as.

## 3 Results and Discussion

### 3.1 2-Thiouracil

Using the adiabatic approach, we simulated trajectories in the D_0_ cationic ground state using initial excitation (photon) energies *E*_*hν*_ of 12–16 eV, in steps of 0.5 eV. With OM2, the 2-TU ionization potential is ∼8.73 eV, in good agreement with experiment.^24,48^ This corresponds to kinetic excess energies in the order of 3.3–7.3 eV. (Note that the IP changes also slightly with sampled initial geometry, with the standard deviation being ∼0.1 eV.) In 2-TU, the sulfur atom is attached to the carbon atom present between two nitrogens, at position 2 (see Figure 1a). We observed fragmentation patterns and abundances produced by the molecular ion during the simulations.

#### 3.1.1 Abundance and Mass Spectra as a Function of Photon Energy; Breakdown Diagram

In this subsection, we first report all fragments observed independent of their status of their *neutral* or *cationic* nature. After that, we will detail explicitly which fragments we consider to be *cationic* in nature.

A tree diagram for 2-TU showing the different fragment possibilities and how different fragments appear at different energies is shown in Figure 2. In addition, images of all fragments for various photon energies are provided in Figure S1.

**Figure 2:**
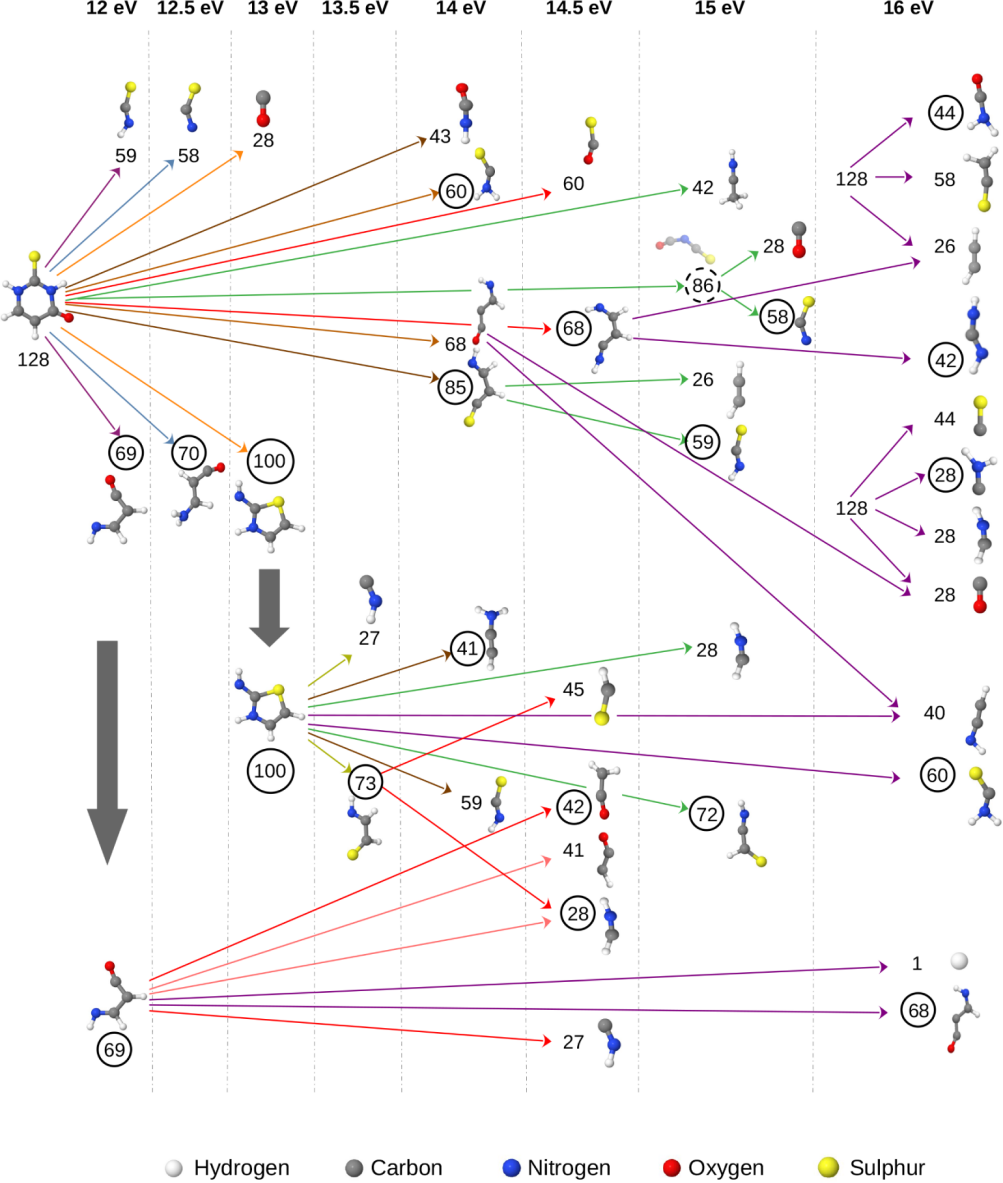
Different possibilities of fragments originating at energies from 12 to 16 eV for 2-thiouracil. The tree diagram illustrates the first occurrence of fragments (cationic and neutral) at various energy levels, highlighting the different possible fragmentations. The numbers in the circles indicate the cationic fragment masses. The dotted circle denotes transient cationic fragments that did not survive the entire simulation. The shown geometries correspond to *t* = 10 ps. White = H, gray = C, blue = N, red = O, yellow = S. Arrow color encodes the energy at which the fragments appear.

Using 12 eV as the lowest energy for photodissociation, we observed two fragments with masses of 59 amu (SCNH) and 69 amu (C_3_NH_3_O), in addition to the parent ion at 128 amu. Relative abundances corresponding to fragment masses are displayed in Figure 3a, which includes both neutral and cationic fragments.

**Figure 3:**
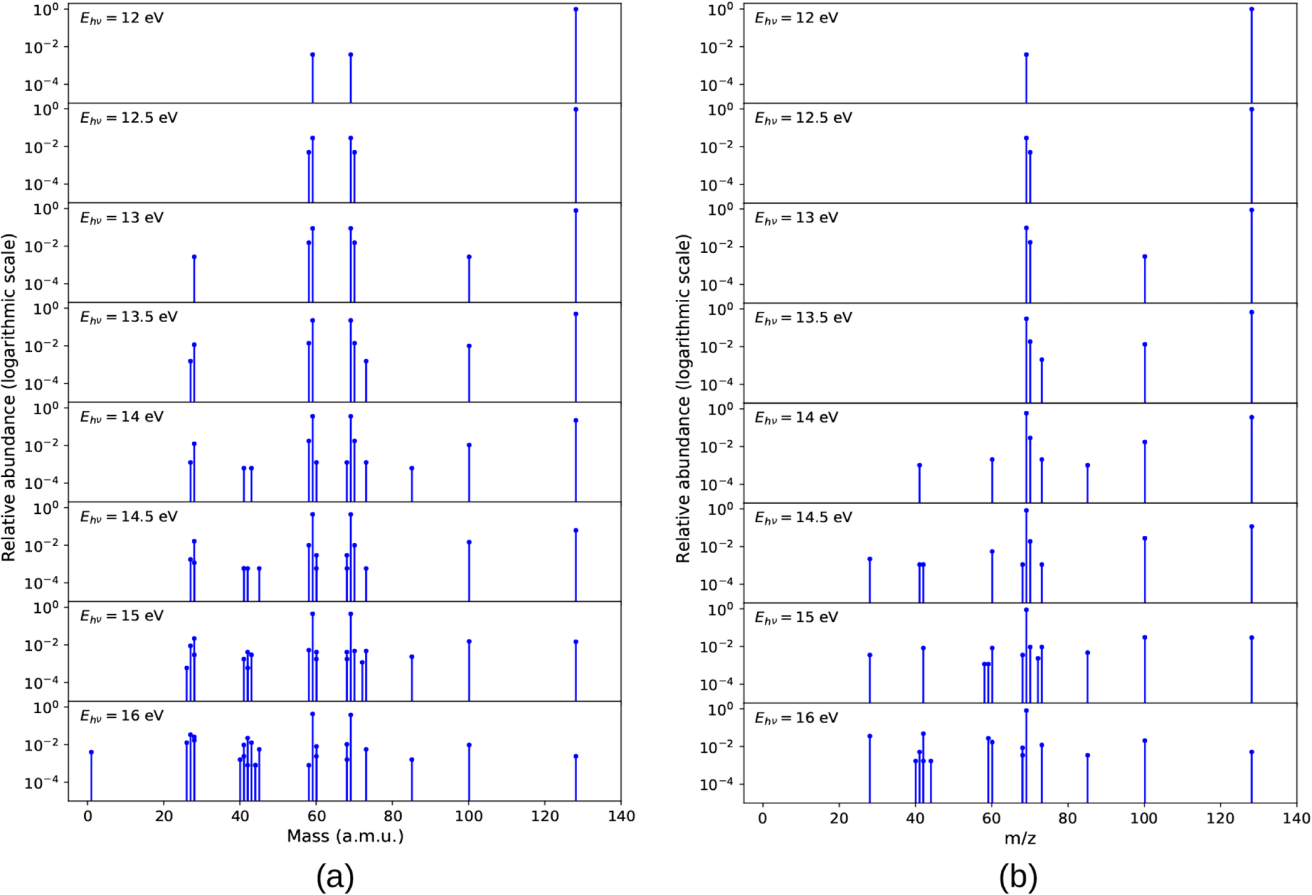
Relative abundance of fragments for various photon energies for 2-thiouracil is depicted as follows: (a) all produced fragments including both the neutral and cationic species and (b) solely cationic fragments post-dissociation.

Increasing the excitation energy by 0.5 eV led to an additional dissociation channel producing fragments with masses of 58 amu (SCN) and 70 amu (C_3_NH_4_O). At 13 eV, the relative abundances of previously identified fragments increased, and two additional fragments with masses of 28 amu (CO) and 100 amu (C_3_N_2_H_4_S) were observed. This trend continued at 13.5 eV, where we identified two more fragments with masses of 27 amu (NCH) and 73 amu (C_2_NH_3_S), where these fragments are produced from the further dissociation of 100 amu (C_3_N_2_H_4_S) fragment. It is evident that as excitation energy increases, both the relative abundances and the diversity of the observed fragments also increase.

At 14 eV of energy, the number and variety of fragments have drastically increased compared to 13.5 eV. Here, we found a new fragment species arose from a different dissociation channel. The 100 amu fragment was dissociated into smaller fragments as 41 amu (C_2_NH_3_) and 59 amu (SCNH). And two new fragments from another new dissociation channel were 43 amu (OCNH) and 85 amu (C_3_NH_3_S). We also observed a fragmentation of the parent ion to 60 amu (SCNH_2_) and 68 amu (C_3_NH_2_O).

In the case of 14.5 eV, some additional fragments arose, where the largest contribution comes from the dissociation of 69 amu fragment. This leads to the formation of new neutral and/or cationic fragments. Here, two distinct types of 41 amu fragments were generated, specifically 41.03 amu (C_2_OH) and 41.05 amu (C_2_NH_3_). Two more new fragments were also found, 28 amu (exactly 28.03 amu (HCNH), different from the previously observed 28.01 amu (CO)) and 45 amu (CSH). Moreover, we found 69 amu (C_3_NH_3_O) → 27 amu (NCH) + 42 amu (C_2_H_2_O) fragmentation and 128 amu (parent) → 60 amu (SCO) + 68 amu (C_3_H_4_N_2_) fragmentation at 14.5 eV. For 15 and 16 eV energies, additional fragmentations were found as detailed in Figure 2.

Considering *cationic* fragments only (determined based on Stevenson’s rule), for the 12 eV energy, during a simulation period of 10 ps, the only cationic fragment observed apart from the parent ion was 69 amu. The overall cationic fragment plot is given in Figure 3b and can be interpreted as a mass spectrum (in which only charged species are detectable). In addition, the identified cations and their relative abundances are collected and provided in Table S1.

As we increased the energy by 0.5 eV, we observed one more cationic fragment of 70 amu. For 12 and 12.5 eV, the 69 amu cationic fragment was not very abundant, but as we increase the photodissociation energy, the trend of decrease in parent fragment can be seen as soon as we hit the molecule with 13 eV photons. In the case of 13 eV, 69 amu abundance is ∼10%. In the case of 13.5 eV, the number has increased 3-fold. Apart from 69 and 70 amu fragments, one more fragment of 73 amu (C_2_NH_3_S) was observed at 13.5 eV of energy, although with a very low abundance of around 0.2%. In the simulation of lesser energy packets (≤13.5 eV), the most abundant cation was the parent one (128 amu).

As the energy is further increased, the number of observed fragments also increased. At 14 eV, besides the 69 amu fragment, we detected the 70 amu fragment (C_3_NH_4_O) with a relative abundance of ∼3% and the 100 amu fragment (C_3_N_2_H_4_S) with ∼2% abundance. The 69 amu fragment accounted for ∼60% of the fragments. Additionally, several other cationic fragments were observed with lower abundances (see Table S1). The parent cation was the second most abundant, contributing approximately ∼36% of the fragments.

The mentioned higher-energy trends continue. Notably, during the 14.5 eV simulation, we observed for the first time that a 28 amu fragment (HCNH) was acting as a cation, although with a very low abundance (0.2%). The most abundant cation was 69 amu with ∼83% abundance. Several new cations were also observed, as detailed in Table S1.

At 15 eV, the most abundant cation was again 69 amu with an abundance of ∼89%. The other most contributing fragments were 100 amu with 3.1% of fragments and 128 amu (parent cation) with 3.0% of fragments. At an energy of 15 eV, we observed that the 59 amu fragment also appears as a cation. In the previous simulations with smaller excitation energies, this fragment was a major neutral dissociation product of the parent cation, formed in conjunction with 69 amu cationic fragment. The 59 amu cation observed at high photon energies emerged through a distinct fragmentation channel―59 amu forms from cationic 85 amu (relative abundance of 0.1%).

At 16 eV, for the first time we saw a decrease in relative abundance of 69 amu, although the most abundant cation remained the 69 amu fragment, but the relative abundance was lower compared to 15 eV. At this energy, the 69 amu fragment exhibited an abundance of 80.7%. The drop in the abundance of the 69 amu fragment comes with an increase in abundances of 42 amu (C_2_H_2_O) to 4.8% and 28 amu (HCNH) to 3.6% as well as appearance of 68 amu (C_3_NH_2_O, H loss from 69 amu) with 0.9%.

Finally, we converted the energy-resolved mass spectra of 2-TU into the breakdown diagram, including the major cations (Figure 4a). The plot shows the correlated rise of 69 amu fragment and decay of the parent cation. The other fragments also show an increase in abundance with an increase in energy, and the bigger fragments are fragmented into smaller fragments with higher photon energy. Overall, the fragments other than 69 amu have a low contribution to the calculated breakdown diagram.

**Figure 4:**
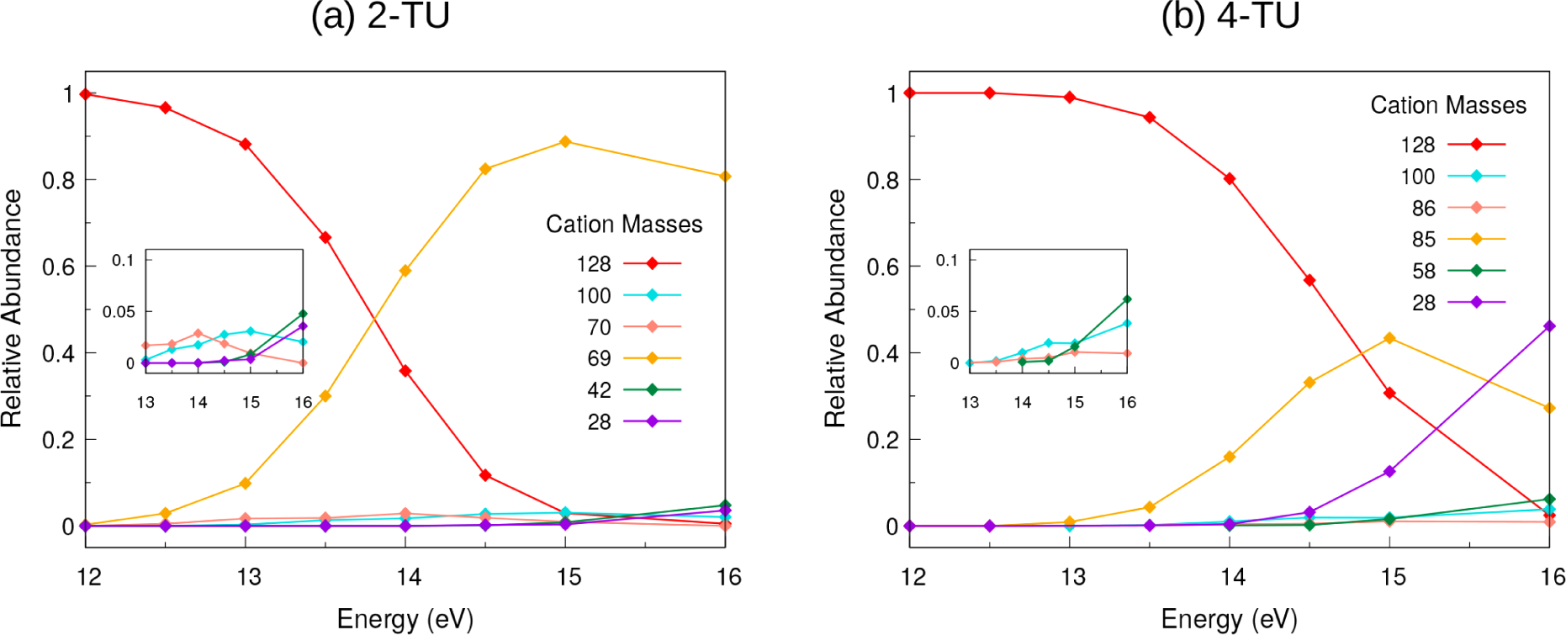
Breakdown of cations under different excitation energies for 2-thiouracil (a) and 4-thiouracil (b). Insets zoom in on the less abundant masses.

#### 3.1.2 Tautomers and First Comparison to Experiment

In our MD simulations for 2-TU, we have not observed the 95 amu fragment which was detected experimentally following VUV and UV multiphoton ionization in ref. 24. This fragment is expected to correspond to the loss of SH (33 amu), and thus should have the formula C_4_H_3_N_2_O. It is known that tautomers of 2-TU containing thiol (SH) may be formed by UV light excitation.^49,50^ Assuming that tautomer formation may be induced by VUV photoionization as well (see the sketch in Figure S2), one could anticipate that the formation of the 95 fragment results from the fragmentation of a tautomeric form. We note that such a tautomerization pathway was observed in glycine.^51^ Therefore, we studied three tautomeric forms of 2-TU, shown in Figure 5, first having the hydrogen transfer from N(1) to sulfur (Figure 5 II). The second tautomeric form has the hydrogen transfer from N(3) to sulfur (Figure 5 III). The third tautomer involves the transfer of hydrogen atoms from both nitrogen atoms, with one transferring to sulfur and the other to oxygen (Figure 5 IV).

**Figure 5:**
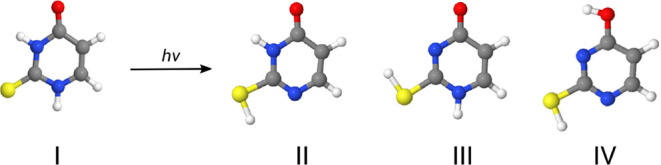
Tautomers of 2-thiouracil.

We now performed BOMD simulations for tautomeric forms of 2-TU (ionizing a tautomer) to see if the 95 amu fragments are formed in MD simulations. 100 trajectories were simulated for each tautomer. Indeed, we observed the SH loss (corresponding to a signal at 95 amu in Figure 6, shown in red sticks). Figure 6 presents the data corresponding to the first two tautomeric forms analyzed (tautomers II and III; neutral and cationic fragments are both shown in Figure 6). The MD simulations for tautomer IV reveal a lower relative abundance of the 95 amu fragment (see Section S2 and Figure S4).

**Figure 6:**
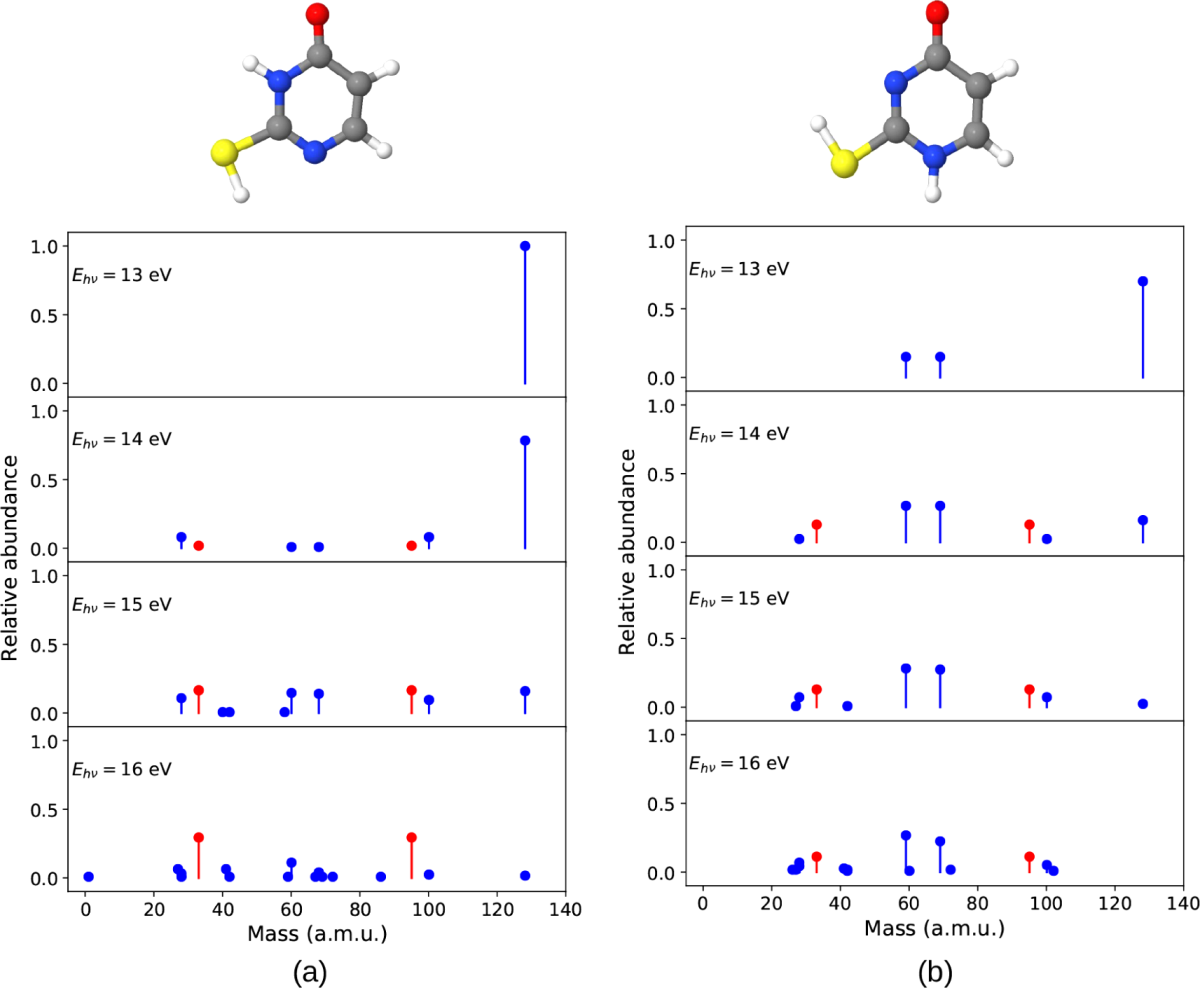
Relative abundance of fragments of tautomers II (a) and III (b) of 2-thiouracil for various photon energies is shown, with red sticks indicating fragments 33 and 95 amu.

We also investigated the energetics of the various tautomers to determine which tautomer is more likely to be thermodynamically stable. The calculated energetics of the tautomers are provided in Section S2, with detailed energetics of the tautomers illustrated in Figure S6. Additionally, the transition state associated with tautomer II formation is depicted in Figure S7 . In the Supporting Information, it is found that in the neutral ground state, the most stable form is tautomer I, followed by tautomer II (the most stable conformer ∼0.55 eV higher in energy), tautomer IV (∼0.58 eV higher), and tautomer III being the least stable one (the most stable conformer being ∼0.98 eV above tautomer I). This is in agreement with an earlier report.^52^ We also note that the same trend in energetics is observed for the cationic ground state (Figure S6).

In Figure 6, the first red stick corresponds to the neutral fragment of 33 amu (SH), and the second red stick corresponds to the cationic fragment with 95 amu (C_4_H_3_N_2_O^+^). The tautomer II (left) generates a greater number of 95 amu fragments compared to the tautomer III (right), at least at higher photon energies. An alternative explanation for the 95 amu fragment would be a separate loss of the sulfur atom and one hydrogen atom. However, this pathway was not observed in our simulations. We finally mention that in the EI-MS spectrum of 2-TU, a signal at 95 amu appears, however, with a relatively low intensity.^53^

#### 3.1.3 Reaction Mechanisms

From the analysis of the trajectories, the following main reaction mechanisms for the formation of major fragments in the case of 2-TU can be extracted:

**69** amu (C_3_NH_3_O^+^) corresponds to SC(2)­N(3)H loss. The mechanism is (i) C(2)–N(1) bond-breaking and (ii) C(4)–N(3) bond-breaking.

**70** amu (C_3_NH_4_O^+^) corresponds to SCN loss. The mechanism is (i) C(2)–N(1) bond-breaking, (ii) C(4)–N(3) bond-breaking, and (iii) H-transfer from N(3) to N(1) or, alternatively, first (iii), then (ii).

**100** amu (C_3_N_2_H_4_S^+^) corresponds to CO loss. The mechanism is (i) C(4)–N(3) bond-breaking, (ii) formation of five-membered ring (either making C(5)–S or C(5)–N(3)), and (iii) C(5)–C(4) bond-breaking.

**28** amu (HCNH^+^) forms from 69 by breaking the C(5)–C(6) bond.

**42** amu (C_2_H_2_O^+^) forms from 69 by the C(5)–C(6) bond-breaking with H-transfer from C(6) to C(5) (CNH neutral), or from N(1) to C(5) (NCH neutral). Few trajectories show the formation of C_2_NH_4_^+^ (42 amu). It begins with breaking C(5)–C(4) bond, then H-transfer from N(3) to C(5), then N(1)–C(2) bond-breaking, then H-transfer from C(6) to C(5).

**41** amu (C_2_NH_3_^+^) forms either from 69 (at 16 eV) or from 100 (at 14 and 14.5 eV). When from 69 this may involve hydrogen transfer from C(6) to C(5), then C(4)­O loss. When from 100 it shows first C(4)–N(3) bond-breaking, then H-transfer from C(5) to N(3), then N(3)­H_2_ transfer to C(5), then C(4)O loss, then C(6)–N(1) bond-breaking.

### 3.2 4-Thiouracil

Similar investigations were done for 4-thiouracil (4-TU), cf. Figure 1b. Again, photon energies between 12 and 16 eV were considered. On the OM2 level at OM2 geometry, the ionization potential of 4-TU is 8.54 eV and slightly lower than for 2-TU, so the kinetic excess energy after photoionization is about 3.5–7.5 eV in this case.

#### 3.2.1 Abundance and Mass Spectra as a Function of Photon Energy; Breakdown Diagram

In this subsection (similarly to 3.1.1), we first report all fragments observed independent of their status of their *neutral* or *cationic* nature. After that, we will detail explicitly which fragments we consider to be *cationic* in nature.

A tree diagram for 4-TU showing the different fragment possibilities and how different fragments appear at different energies is shown in Figure 7. In addition, images of all fragments for various photon energies are provided in Figure S8.

**Figure 7:**
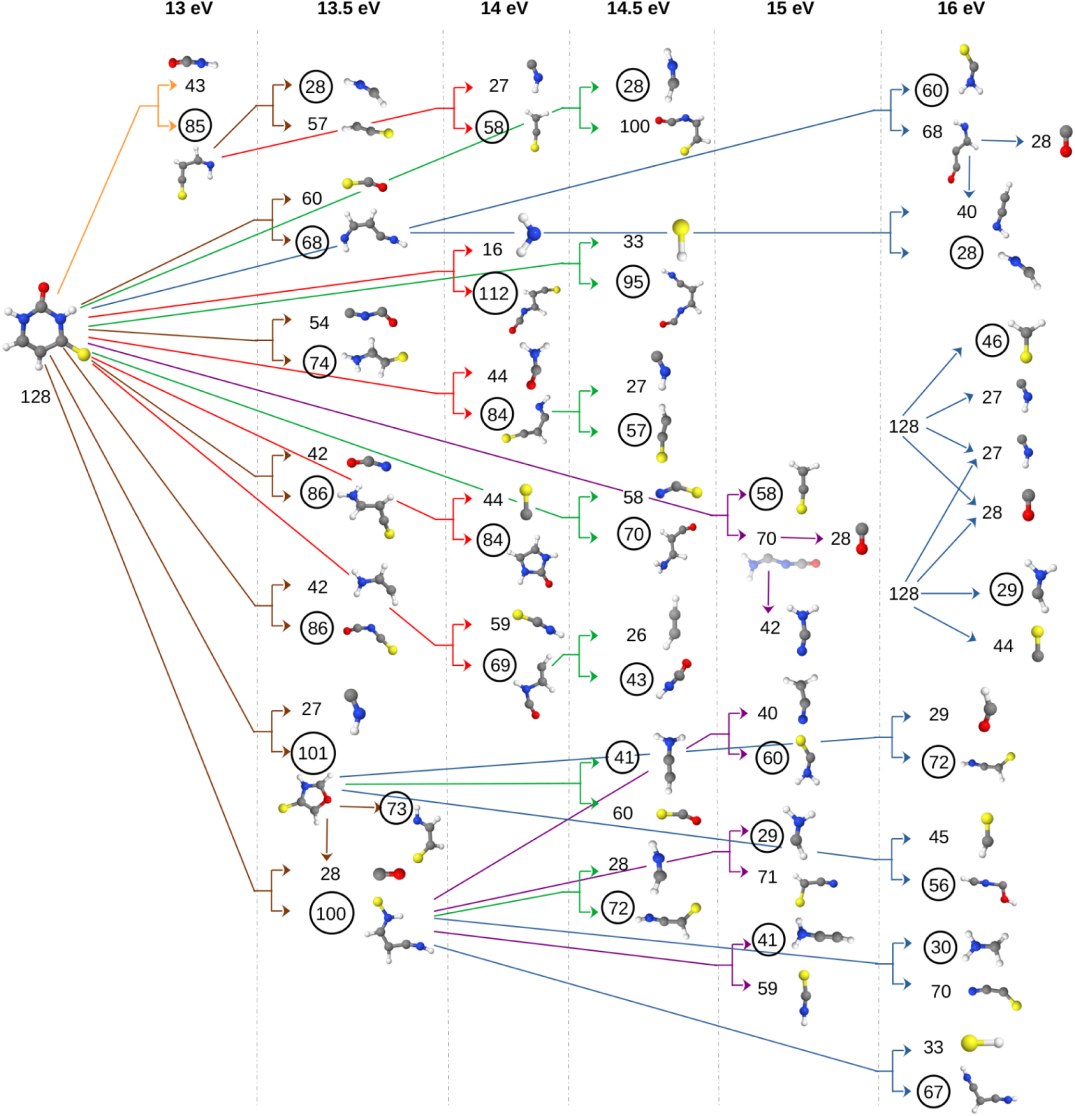
Different possibilities of fragments originating at energies from 12 to 16 eV for 4-thiouracil. The tree diagram illustrates the first occurrence of fragments (cationic and neutral) at various energy levels, highlighting the different possible fragmentations. The numbers in the circles indicate the cationic fragment masses. The shown geometries correspond to *t* = 10 ps. White = H, gray = C, blue = N, red = O, yellow = S. Arrow color encodes the energy at which the fragments appear.

For 4-TU, unlike 2-TU, despite the higher initial kinetic energy, at 12 and 12.5 eV photon energies, no fragmentations were observed, meaning that for the whole simulation period of 10 ps, the parent molecular ion remained intact as seen in Figure 8a.

**Figure 8:**
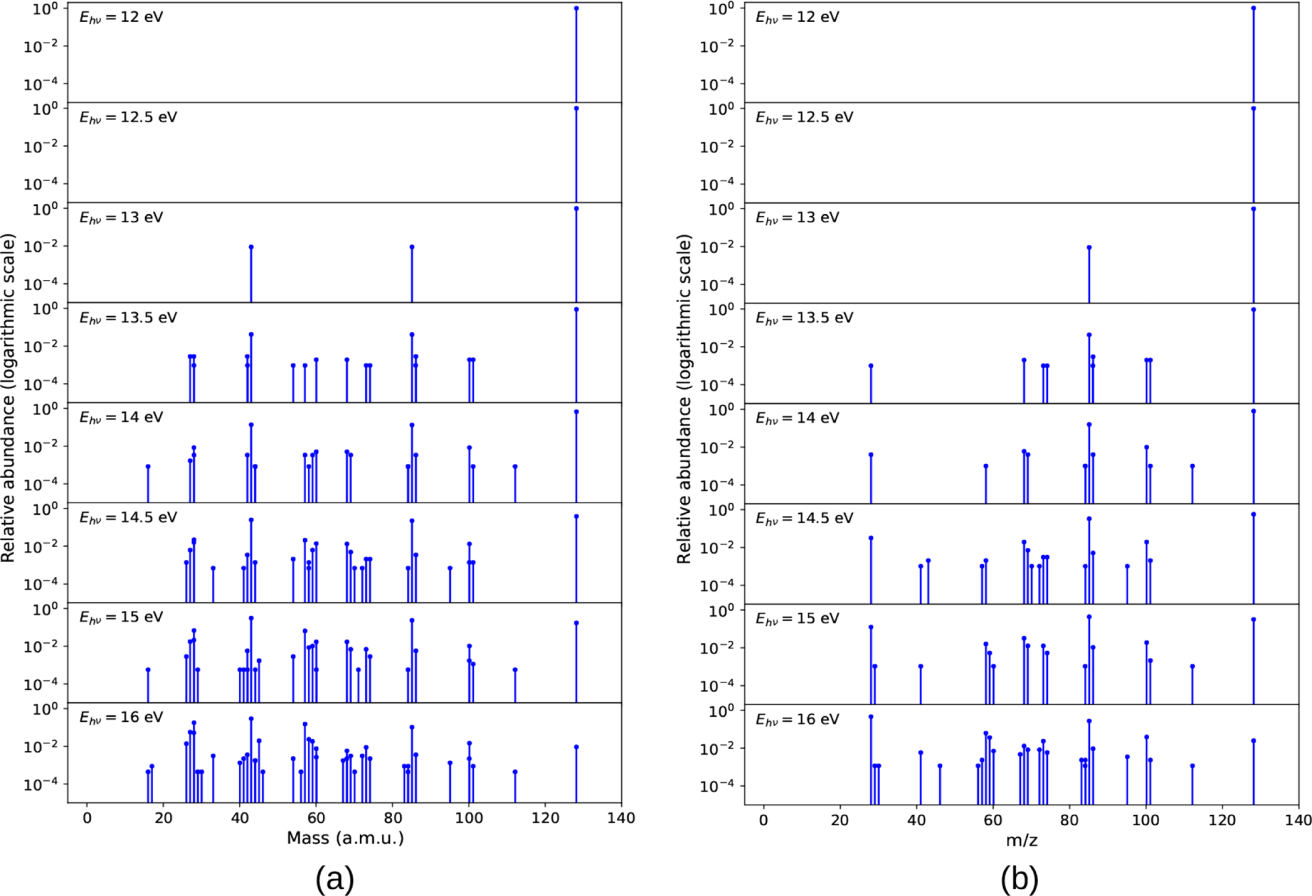
Relative abundance of fragments for various photon energies for 4-thiouracil is depicted as follows: (a) all produced fragments including both the neutral and cationic species and (b) solely cationic fragments after dissociation.

Fragmentations occur at 13 eV and above. At 13 eV, we see two fragments resulting from one type of fragmentation, at mass 43 amu (OCNH) and 85 amu (C_3_NH_3_S). Interestingly, as we increase the energy by just 0.5 eV to 13.5 eV, we observe many more fragments compared to 13 eV, unlike in the case of 2-TU where to observe many different fragmentations, one needs to provide higher energy to the molecule. However, the total amount of 4-TU fragmentation taking place is less compared to 2-TU (cf. Figures 3a and 8a for *E_hν_* = 13.5 eV). The fragments contributing highest to the relative abundance are still 43 and 85 amu fragments (apart from the parent ion), and the percentages of finding these fragments are also slightly larger than those for 13 eV photon energy. Some smaller fragments were also found, such as 27 amu (NCH) and two types of 28 amu (HCNH and CO). Other detected fragments with relatively high abundance are 42 amu (CNO), 60 amu (CSO), 68 amu (C_3_H_4_N_2_), 86 amu (C_3_H_4_NS), 100 amu (C_3_H_4_N_2_S), and 101 amu (C_3_H_3_NSO).

At 14 eV, several different fragments were observed compared to 2-TU. Notably, a fragment with 16 amu (NH_2_) was detected for the first time, albeit with a very low relative abundance. The major fragments here were again 43 and 85 amu. Increasing the energy to 14.5 eV produces similar types of fragments with relatively higher individual abundance and decreasing abundance of parent ion. At 15 eV, the abundance of the parent ion is no longer the largest one. The parent cation has been fragmented into many smaller neutral and cationic fragments. The major contributors to relative abundances here are fragments 43 and 85 amu. At 16 eV, we see that the contribution from the molecular ion (128 amu) in the relative abundance plot has completely been diminished and, moreover, the major contributing bigger fragment 85 amu has also been fragmented into more smaller fragments which leads to the lesser contribution from 85 amu to the relative abundance plot.

The photon-energy dependent mass spectra are shown in Figure 8b, and all identified *cations* and their relative abundances are collected in Table S1. For 12 and 12.5 eV, we see only the parent cation. At 13 eV, the only cation that we observe (apart from the parent ion) was fragment 85 amu with a relative abundance of 0.9%. At 13.5 eV, even more cationic fragments were observed with the largest signals corresponding to the parent ion with 94.4% and fragment 85 amu with a relative abundance of 4.3%. Less abundant cations range from 28 amu (HCNH) to 101 amu (C_3_H_3_NSO) as detailed in Table S1. Although the number of cationic fragments found was higher (10 fragments), the individual abundances of most fragments were small in the case of 13.5 eV.

At 14 eV, the major fragments in the mass spectrum were 85 amu with a relative abundance of 16.0% and molecular cation with a relative abundance of 80.7% followed by fragment 100 amu (C_3_H_4_N_2_S) with a relative abundance of ∼1%. All other cationic fragments found here have very low abundances (see Table S1). In the case of 14.5 eV, fragment 85 amu has more relative abundance compared to lower energies, with 33.1% contribution in the overall relative abundance. The parent cation has a relative abundance of 56.7%. Additional relatively big fragments with relatively large contribution are 100 amu (C_3_H_4_N_2_S) and 68 amu (C_3_H_4_N_2_), each with 1.9% of abundance. Now, as the energy has increased, the abundances of smaller fragments have also increased. Specifically, the cationic fragment of 28 amu (HCNH) has a relative abundance of 3.2%. Other fragments have lesser abundances (Table S1).

At 15 eV, the contribution from molecular cation decreased to 32% and fragment 85 amu rose to a relative abundance of 43.4%, thus surpassing the parent. The smallest cationic fragment 28 amu (HCNH) has been formed the most number of times (compared to other smaller fragments) and has a relative abundance of 12.6%. Some other fragments can also be seen formed more frequently, e.g., 68 amu (C_3_H_4_N_2_) has a relative abundance of 3.2%, see Table S1.

At 16 eV, the most abundant cation is fragment 28 amu with a relative abundance of 46.1%, followed by the cation 85 amu with 27.2%, and the molecular cation has been diminished to 2.5%. The types of smaller cationic fragments have increased, meaning a bigger fragment has been dissociated into many smaller fragments (see Figure 7 and Table S1). In total, we identified 26 different cations at 16 eV for 4-TU, which is much larger than for 2-TU (15 cationic species at 16 eV), see Table S1.

Finally, the breakdown diagram for 4-TU is shown in Figure 4b. The major contributions for the cationic fragments come from the fragments 85 and 28 amu. As the excitation energy increases, the decrease in the parent cation is evident, and there is an increase in the abundance of fragment 85 amu. But beyond 15 eV, the cationic fragment 85 amu starts to dissociate into smaller fragments, and the major contribution goes to fragment 28 amu, which, in turn, increases the abundance at higher energies. The parent ion (128 amu) and the fragments 85 and 28 amu contribute the most to the breakdown diagram. All other major cations shown in Figure 4b increase in abundance with the growing energy, but their contribution is overall minor. Comparing 2-TU and 4-TU in their breakdown diagrams, we see clear differences, which will be discussed in more detail in Section 3.3.

#### 3.2.2 Reaction Mechanisms

The reaction mechanisms of formation of major fragments in the case of 4-TU are as follows.

**85** amu (C_3_NH_3_S^+^) corresponds to OC(2)­N(3)H loss. The mechanism is (i) C(2)–N(1) bond-breaking and (ii) C(4)–N(3) bond-breaking.

**86** amu (C_3_H_4_NS^+^) corresponds to OC(2)­N(3) loss. The mechanism is (i) C(2)–N(1) bond-breaking, then a hydrogen transfer from the N(3) hydrogen to N(1), making the nitrogen at position 1 having two hydrogens, and (ii) breaking of the C(4)–N(3) bond.

**100** amu (C_3_H_4_N_2_S^+^) corresponds to a CO loss. The mechanism is (i) C(2)–N(1) bond-breaking, (ii) sulfur transfer to N(1), (iii) bond-breaking of C(2)–N(3) or, alternatively, first (iii), then (ii). Other dissociative mechanisms may involve the transfer of hydrogen to nitrogen N(1) from either another nitrogen atom or one of the carbon atoms. Following this H transfer, the formation of a five- or six-membered ring could occur. However, the likelihood of these alternative mechanisms is significantly lower compared to the primary mechanism described above.

**28** amu (HCNH^+^) forms from either 85 or 100 amu by breaking the C(5)–C(6) bond.

**58** amu (C_2_H_2_S^+^) forms from 85 by breaking the C(5)–C(6) bond and then involves a hydrogen transfer from C(6) to C(5).

**68** amu $\left(C_{3}H_{4}N_{2}^{+}\right)$ corresponds to SCO loss. The mechanism is (i) breaking of the C(2)–N(3) bond initiates the process, (ii) subsequently, sulfur approaches the C(2) carbon, which leads to the cleavage of the bond S–C(4), resulting in the departure of the SCO.

### 3.3 Further Discussion

In the simulations for both molecules, we observed that as the energy increases, the number of fragments also increases. Although both molecules appear quite similar, the most abundant fragments are very different from each other. For 2-TU, as previously discussed, the primary contributing fragments were 59 amu (SCNH) and 69 amu (C_3_NH_3_O). In contrast, for 4-TU, the most significant fragments were 43 amu (OCNH) and 85 amu (C_3_NH_3_S). Remarkably, this corresponds to breaking the same bonds, C(2)–N(1) and C(4)–N(3) in both isomers (cf. Figure 1). In fact, the C–N single bond is the one with the lowest dissociation energy of all bonds within both molecules (330 kJ/mol, compared to 368 kJ/mol for C–C, for example).^54^

Another common observation in both simulations is that with increasing energy, the relative abundance of a smaller fragment, specifically 28 amu (HCNH), consistently increased. We also observed that above a certain energy, larger fragments dissociate into smaller fragments. In the case of 2-TU, fragment 69 amu has always increased its relative abundance until 15 eV of excitation, after which the fragment dissociates into smaller fragments, mostly leading to the generation of cationic fragments 28 amu (HCNH) and 42 amu (C_2_H_2_O). A similar situation was observed for 4-TU as well where fragment 85 amu after an energy of 15 eV decreases substantially and leads to more formation of cationic fragments 28 amu (HCNH) and also 58 amu (C_2_H_2_S) (with a lower abundance).

However, there are also clear differences between 2-TU and 4-TU. Looking at Table S1, we see that 4-TU yields more fragments than 2-TU for *E_hν_* ≥ 13.5 eV (albeit mostly with low abundances). At the same time, the breakdown diagrams of Figure 4 show that the falloff of the parent cation abundance is shifted to higher energies for 4-TU. This is despite the fact that more kinetic energy is being deposited into 4-TU compared to 2-TU following absorption of the same photon energy because of a lower ionization potential of 4-TU (see above). Thus, sulfur in position 4 makes 4-TU more resistive to fragmentation than 2-TU.

The average time required to dissociate 2-TU^+^ to form fragments 59 and 69 amu is ≤5 ps across the smaller excitation energies (12–13.5 eV), while for higher excitation energies (14–16 eV), it is ≤3.5 ps. The subsequent formation of the 28 amu fragment occurs within the next 1–2 ps. A similar trend can also be seen in 4-TU, where the average time required to dissociate into fragments 43 and 85 amu is ≤4 ps for low excitation energies, while for higher excitation energies, it takes ≤2.5 ps to dissociate the parent cation. This phenomenon can be explained by considering that at higher excitation energies, the molecule has enough energy to rapidly overcome the bond dissociation energy. Additionally, a higher excitation energy grants access to a larger number of dissociation channels. Furthermore, methods like disruptive probing could be used to experimentally quantify these lifetimes.^55^

### 3.4 Comparison to Experiment

In this subsection, we compare our calculations for 2-TU with the (photon-energy resolved) experimental mass spectra reported in ref. 24 in more detail.

The simulations conducted in the present work produced principal cationic fragments of 69 amu for 2-TU, findings that are consistent with the experimental results reported by Robinson et al.^24^ Another fragment that was predominantly seen in the experiment was 28 amu. In our simulations, this fragment was also generated; however, it was produced in relatively lower abundances. Furthermore, the experiment^24^ reports fragments at 41, 42, 58, 60, 70, and 100 amu, although in lower abundance; our simulations similarly indicated reduced abundances for these fragments. For fragments 41 and 42 amu, consistent with the analysis of Robinson et al.,^24^ our simulations confirmed that the most abundant species for fragment 41 amu was $C_{2}NH_{3}^{+}$ , while for fragment 42 amu, the primary species was $C_{2}H_{2}O^{+}$ .

Comparing the energetics of fragmentation, specifically our calculated breakdown diagram for 2-TU (Figure 4a) with Figure 2a of ref. 24, we observe that the calculated abundance curves are shifted to higher energies. Figure 2a of the experiment^24^ shows a photoionization spectrum and contains the ionization matrix elements, in contrast to the theoretical breakdown spectrum. However, one can discern that the first breakdown from parent ion to fragment 69 happens at lower energies in the experiment, showing a dominant 69 fragment with no parent already around ∼11.5–12 eV. In the calculated diagram, the crossing point is at ∼13.8 eV (see Figure 4a). The energy disparity between experiment and theory is also observed for other fragments, e.g., 70 and 100 amu.

There are (at least) two factors responsible for this discrepancy. First, as already mentioned in Section 2, we neglected the initial kinetic energy of the neutral (∼0.58 eV), *vide supra*. Therefore, the “computational” photon energy of 12 eV will translate to 11.42 eV. Second, it might well be that 10 ps is not enough to capture all fragmentation events, especially at lower photon energies. The nonsufficient theoretical time window thus might increase the relative abundance of the parent ion in the simulation with regard to the experiment. To test this, we simulated 200 trajectories at *E_hν_* = 13 eV for 100 ps. We observed that the parent ion abundance decreased further by a factor of ∼1.7. However, such long time scales are computationally demanding, and it is thus challenging to perform proper statistics for long propagation times.

### 3.5 Nonadiabatic Surface Hopping (NASH) Calculations

Finally, we conducted test simulations of the nonadiabatic dynamics for the example of 2-TU^+^ to investigate the involvement of excited cationic states in the post-ionization dynamics of the parent molecule. In our test calculations, our main objective was not so much the fragmentation itself but to test the hypothesis/assumption of a rapid internal conversion of higher doublet states of the cation, to the ground state, D_0_. Exemplarily, we focused on 2-TU. We note that ultrafast internal conversion has been observed/predicted in other molecular cations.^39,56,57^

We used Tully’s surface hopping approach^45^ combined with the ROOM2/CISD method, employing an active space of 5 highest occupied and 5 lowest virtual orbitals (denoted as 5 × 5). We performed surface hopping simulations over a 1 ps time scale, initiating excitations to states D_2_, D_6_, and D_8_, selected due to their relatively high oscillator strengths provided in Table 1. We note that this choice corresponds to the absorption of the cation (D_0_ → D_*i*_) rather than photoionization of the neutral (S_0_ → D_*i*_). For the latter, one could calculate Dyson orbitals to estimate photoionization cross sections.^58^ In fact, we have recently employed this approach for thiouracils at the EOM-IP-CCSD level, and the Dyson norms were found to be approximately the same for the lowest 11 transitions (S_0_ → D_0_ ... D_10_).^59^ Thus, the present choice of D_2_, D_6_, and D_8_ can be viewed as merely a test for various initial excitation energies. The initial conditions (i.e., initial geometries and nuclear velocities) were sampled using the Wigner distribution (for the D_0_ state) at a temperature of 0 K.^60^ Again, we note that sampling in the S_0_ neutral state would be more appropriate for modeling photoionization experiments. The associated kinetic energy (arising from the initial velocities) was approximately 1.07 eV, leading to total energies of 2.9 eV for D_2_, 5.4 eV for D_6_, and 5.8 eV for D_8_, referenced from the D_0_ state. To relate these energies to the S_0_ state, an ionization potential (IP) of ∼8.7 eV (as discussed above) must be added. This adjustment results in energies ranging from 11.6 to 14.5 eV. These values are consistent with the energy range used in our BOMD calculations. For each initial state, 100 independent trajectories were simulated focusing on (potential) fragmentation pathways and excited-state lifetimes.

**Table 1:** Excitation Energies *E* (in eV) and Oscillator Strengths *f* for the First Ten Transitions of the 2-TU Cation Calculated at the ROOM2/CISD(5 × 5) Level at the Optimized UB3LYP/6-31G* Geometry

Transition	*E* (eV)	*f*
D_0_ → D_1_	0.143	0.000
D_0_ → D_2_	1.862	0.025
D_0_ → D_3_	2.579	0.000
D_0_ → D_4_	3.768	0.000
D_0_ → D_5_	3.822	0.002
D_0_ → D_6_	4.368	0.030
D_0_ → D_7_	4.421	0.000
D_0_ → D_8_	4.754	0.062
D_0_ → D_9_	4.852	0.000
D_0_ → D_10_	5.079	0.001

In all cases (D_2_, D_6_, and D_8_), no fragmentation was observed when using the 4 Å bond distance threshold, while relaxation to the ground state occurred consistently on an ultrafast time scale. The ground-state-recovery time constant τ was calculated using a single-exponential fit, $P_{D_{0}}=1-\exp\left(-t/\tau \right)$ . Relaxation to the cationic ground state when starting in state D_2_ takes around ∼500 fs. For D_6_, the relaxation time was around 800 fs, while for D_8_, ∼63% of the population transitioned to the ground state (D_0_) in around 900 fs. The absence of fragmentation could be attributed to insufficient time to induce dissociation. In fact, as argued in Section 3.3, typical fragmentation times are several picoseconds, i.e., significantly longer than the relaxation times to D_0_. It is therefore plausible that for the investigated molecules, dissociation takes place following internal conversion, mainly in the cationic ground state, D_0_.

We also applied a weaker bond-breaking criterion of 2 Å to further investigate the possible onset of fragmentation. We found that in the case of D_8_, 5 trajectories out of 100 showed dissociation into the major fragments corresponding to the 59–69 fragment channel also observed in the case of the BOMD simulations. In all these cases, too, the fragmentation occurred after relaxation to the cationic ground state.

Finally, we note that no tautomerization was observed in our NASH trajectories. Apparently, one would need to simulate many more NASH trajectories and potentially test various electronic structure methods to elucidate the hypothesized photoinduced tautomerization in cationic states, which goes beyond the scope of the present work and should be addressed in a separate study.

## 4 Summary and Conclusions

In this work, we examined the VUV-induced fragmentation patterns of thiouracil derivatives, specifically 2-thiouracil and 4-thiouracil, and compared their fragmentation products obtained from the simulations using a BOMD approach. Additionally, we compared our simulated fragmentation products of 2-thiouracil against experimental results previously reported by Robinson et al.^24^ finding that the fragments predicted in our simulations were consistent with experimental observations, where the highest contributing cationic fragment was 69 amu (C_3_NH_3_O^+^, SCNH loss).

We also noted the absence of certain fragments, particularly the 95 amu fragment, in our simulations for the primary tautomer (I) expected in the gas phase. We showed that by starting with alternative tautomers in the gas phase, the 95 amu fragment (C_4_H_3_N_2_O^+^, SH loss) was observed with significant abundance in our BOMD simulations. While we expect that tautomerization may be initiated on the electronic excited state of the cationic system, unfortunately, our test NASH simulations did not provide any evidence of tautomerization taking place, nor subsequent fragmentation to produce the 95 amu ion. This may be due to limitations of the simulation performed here as part of this work, and further theoretical work is needed to better understand the differences between theory and experiment. However, observation of the 95 amu fragment in our BOMD simulations for tautomers other than I indicates that tautomers can play a significant role in shaping the overall fragmentation profile.

We compared the highest fragmentation products of 2-TU with 4-TU. The major contributing cationic fragments to the mass spectrum in 4-TU were 85 amu (C_3_NH_3_S^+^, OCNH loss) and 28 amu (HCNH^+^). The dissociation channel to produce the most abundant fragment was the same in both the derivatives of thiouracil, specifically the same bonds [C(2)–N(1) and C(4)–N(3)] were broken in both molecules to form the most abundant cations (69 amu in the case of 2-TU and 85 amu in 4-TU). The dominant fragmentations follow breaking the weakest (C–N) bonds in the molecules. However, sulfur in the 4 position seemingly protects the ring from fragmentation, compared to when it is in the 2 position, as follows from the blue-shifted falloff of the parent ion abundance despite the lower ionization potential of 4-TU. The formation of 28 amu was predominantly seen after 14.5 eV of energy, resulting from the further fragmentation of 85 amu at elevated energies.

To broaden our investigation of ultrafast fragmentation and dynamics and account for nonadiabatic effects, we conducted preliminary surface hopping calculations at the ROOM2/CISD(5 × 5) level for 2-TU^+^. Our simulations reveal that internal conversion occurs within an ultrafast time scale for 2-thiouracil, confirming the frequently used hypothesis that fragmentation takes place in the cationic ground state.

In summary, the MD simulations presented herein provide detailed mechanistic insights into VUV-induced dissociative photoionization of thiouracils, providing a roadmap for analogous simulations of similar molecules in the future.

## Supplementary Material



## Notes

Photodissociative fragments of 2-TU^+^; tautomers of 2-TU; photodissociative fragments of 4-TU^+^; relative abundances of cations at different photon energies for 2-TU^+^ and 4-TU^+^; optimized geometries of 2-TU and 4-TU; and SH simulations for 2-TU^+^ (PDF)

## References

[1] Anderson G. W., Halverstadt I., Miller W. H., Roblin R. O. (1945). Studies in Chemotherapy.
X. Antithyroid Compounds. Synthesis of 5-and 6-Substituted 2-Thiouracils
from *β*-Oxoesters and Thiourea1. J. Am. Chem. Soc..

[2] Prachayasittikul S., Worachartcheewan A., Nantasenamat C., Chinworrungsee M., Sornsongkhram N., Ruchirawat S., Prachayasittikul V. (2011). Synthesis
and structure–activity relationship of 2-thiopyrimidine-4-one
analogs as antimicrobial and anticancer agents. Eur. J. Med. Chem..

[3] Dencker L., Larsson B., Olander K., Ullberg S. (1982). A new melanoma
seeker
for possible clinical use: Selective accumulation of radiolabelled
thiouracil. Br. J. Cancer.

[4] Milder S. J., Kliger D. S. (1985). Spectroscopy and
photochemistry of thiouracils: implications
for the mechanism of photocrosslinking in tRNA. J. Am. Chem. Soc..

[5] Zou X., Dai X., Liu K., Zhao H., Song D., Su H. (2014). Photophysical
and photochemical properties of 4-thiouracil: time-resolved IR spectroscopy
and DFT studies. J. Phys. Chem. B.

[6] Navarrete-Miguel M., Giussani A., Rubio M., Boggio-Pasqua M., Borin A. C., Roca-Sanjuán D. (2024). Quantum-Chemistry
Study of the Photophysical
Properties of 4-Thiouracil and Comparisons with 2-Thiouracil. J. Phys. Chem. A.

[7] Mayer D., Handrich M., Picconi D., Lever F., Mehner L., Murillo-Sanchez M. L., Walz C., Titov E., Bozek J., Saalfrank P. (2024). X-ray photoelectron
and NEXAFS spectroscopy
of thionated uracils in the gas phase. J. Chem.
Phys..

[8] Pollum M., Jockusch S., Crespo-Hernandez C. E. (2014). 2, 4-Dithiothymine
as a potent UVA
chemotherapeutic agent. J. Am. Chem. Soc..

[9] Reelfs O., Karran P., Young A. R. (2012). 4-thiothymidine
sensitization of
DNA to UVA offers potential for a novel photochemotherapy. Photochem. Photobiol. Sci..

[10] Farrell K. M., Brister M. M., Pittelkow M., Sølling T. I., Crespo-Hernández C. E. (2018). Heavy-atom-substituted
nucleobases
in photodynamic applications: substitution of sulfur with selenium
in 6-thioguanine induces a remarkable increase in the rate of triplet
decay in 6-selenoguanine. J. Am. Chem. Soc..

[11] Uleanya K. O., Dessent C. E. (2021). Investigating the
mapping of chromophore excitations
onto the electron detachment spectrum: photodissociation spectroscopy
of iodide ion–thiouracil clusters. Phys.
Chem. Chem. Phys..

[12] Uleanya K. O., Cercola R., Nikolova M., Matthews E., Wong N. G., Dessent C. E. (2020). Observation of enhanced dissociative photochemistry
in the non-native nucleobase 2-thiouracil. Molecules.

[13] Salet C., Bensasson R., Favre A. (1983). Studies on the triplet excited state
of 4-thiouridine. Photochem. Photobiol..

[14] Mayer D., Picconi D., Robinson M. S., Gühr M. (2022). Experimental
and theoretical gas-phase absorption spectra of thionated uracils. Chem. Phys..

[15] Mai S., Marquetand P., González L. (2015). A static picture of the relaxation
and intersystem crossing mechanisms of photoexcited 2-thiouracil. J. Phys. Chem. A.

[16] Mai S., Marquetand P., González L. (2016). Intersystem crossing pathways in
the noncanonical nucleobase 2-thiouracil: A time-dependent picture. J. Phys. Chem. Lett..

[17] Martínez-Fernández L., Granucci G., Pollum M., Crespo-Hernández C. E., Persico M., Corral I. (2017). Decoding the
molecular basis for
the population mechanism of the triplet phototoxic precursors in UVA
light-activated pyrimidine anticancer drugs. Chem. Eur. J..

[18] Pollum M., Jockusch S., Crespo-Hernández C. E. (2015). Increase
in the
photoreactivity of uracil derivatives by doubling thionation. Phys. Chem. Chem. Phys..

[19] Sánchez-Rodríguez J. A., Mohamadzade A., Mai S., Ashwood B., Pollum M., Marquetand P., González L., Crespo-Hernández C. E., Ullrich S. (2017). 2-Thiouracil intersystem crossing photodynamics studied
by wavelength-dependent photoelectron and transient absorption spectroscopies. Phys. Chem. Chem. Phys..

[20] Vendrell-Criado V., Sáez J. A., Lhiaubet-Vallet V., Cuquerella M. C., Miranda M. A. (2013). Photophysical properties of 5-substituted 2-thiopyrimidines. Photochem. Photobiol. Sci..

[21] Yu H., Sanchez-Rodriguez J. A., Pollum M., Crespo-Hernández C. E., Mai S., Marquetand P., González L., Ullrich S. (2016). Internal conversion
and intersystem crossing pathways in UV excited, isolated uracils
and their implications in prebiotic chemistry. Phys. Chem. Chem. Phys..

[22] Gobbo J. P., Borin A. C. (2014). 2-Thiouracil deactivation pathways and triplet states
population. Comput. Theor. Chem..

[23] Teles-Ferreira D. C., Conti I., Borrego-Varillas R., Nenov A., Van Stokkum I. H. M., Ganzer L., Manzoni C., de Paula A. M., Cerullo G., Garavelli M. (2020). A unified
experimental/theoretical description of the
ultrafast photophysics of single and double thionated uracils. Chem. Eur. J..

[24] Robinson M. S., Niebuhr M., Gühr M. (2023). Ultrafast
photo-ion probing of the
relaxation dynamics in 2-thiouracil. Molecules.

[25] Mayer D., Lever F., Gühr M. (2024). Time-resolved
x-ray spectroscopy
of nucleobases and their thionated analogs. Photochem. Photobiol..

[26] Wang Z., Rana T. M. (1996). RNA Conformation in the Tat- TAR Complex Determined
by Site-Specific Photo-Cross-Linking. Biochemistry.

[27] Favre A., Saintomé C., Fourrey J.-L., Clivio P., Laugâa P. (1998). Thionucleobases
as intrinsic photoaffinity probes of nucleic acid structure and nucleic
acid-protein interactions. J. Photochem. Photobiol.
B Biol..

[28] Meisenheimer K. M., Koch T. H. (1997). Photocross-linking of nucleic acids to associated proteins. Crit. Rev. Biochem. Mol. Biol..

[29] Nam Y., Keefer D., Nenov A., Conti I., Aleotti F., Segatta F., Lee J. Y., Garavelli M., Mukamel S. (2021). Conical Intersection Passages of
Molecules Probed by
X-ray Diffraction and Stimulated Raman Spectroscopy. J. Phys. Chem. Lett..

[30] Nam Y., Montorsi F., Keefer D., Cavaletto S. M., Lee J. Y., Nenov A., Garavelli M., Mukamel S. (2022). Time-Resolved Optical Pump-Resonant X-ray Probe Spectroscopy
of 4-Thiouracil: A Simulation Study. J. Chem.
Theory Comput..

[31] Becke A. D. (1993). Density-Functional
Thermochemistry III. The Role of Exact Exchange. J. Chem. Phys..

[32] Stephens P. J., Devlin F. J., Chabalowski C. F., Frisch M. J. (1994). Ab Initio Calculation
of Vibrational Absorption and Circular Dichroism Spectra Using Density
Functional Force Fields. J. Phys. Chem..

[33] Hehre W. J., Ditchfield R., Pople J. A. (1972). Self-Consistent Molecular Orbital
Methods. XII. Further Extensions of Gaussian-Type Basis Sets for Use
in Molecular Orbital Studies of Organic Molecules. J. Chem. Phys..

[34] Hariharan P. C., Pople J. A. (1973). The influence of polarization functions on molecular
orbital hydrogenation energies. Theor. Chim.
Acta.

[35] Francl M.
M., Pietro W. J., Hehre W. J., Binkley J. S., Gordon M. S., DeFrees D. J., Pople J. A. (1982). Self-consistent molecular orbital
methods. XXIII. A polarization-type basis set for second-row elements. J. Chem. Phys..

[36] Frisch, M. J. ; Trucks, G. W. ; Schlegel, H. B. ; Scuseria, G. E. ; Robb, M. A. ; Cheeseman, J. R. ; Scalmani, G. ; Barone, V. ; Petersson, G. A. ; Nakatsuji, H. , Gaussian 16 Revision C.01; Gaussian Inc.: Wallingford CT, 2016.

[37] Weber W., Thiel W. (2000). Orthogonalization corrections
for semiempirical methods. Theor. Chem. Acc..

[38] MNDO2020 is a semiempirical quantum chemistry program written by Thiel, W. , with contributions from Beck, M. ; Billeter, S. ; Kevorkiants, R. ; Kolb, M. ; Koslowski, A. ; Patchkovskii, S. ; Turner, A. ; Wallenborn, E.-U. ; Weber, W. https://mndo.kofo.mpg.de (accessed 3 July 2025).

[39] Roy B., Titov E., Saalfrank P. (2023). Computational
study of the adamantane
cation: simulations of spectroscopy, fragmentation dynamics, and internal
conversion. Theor. Chem. Acc..

[40] Grimme S. (2013). Towards first
principles calculation of electron impact mass spectra of molecules. Angew. Chem., Int. Ed..

[41] Majer K., Signorell R., Heringa M. F., Goldmann M., Hemberger P., Bodi A. (2019). Valence Photoionization of Thymine: Ionization Energies, Vibrational
Structure, and Fragmentation Pathways from the Slow to the Ultrafast. Chem. Eur. J..

[42] Warren R., Dunlap B. (1996). Fractional occupation numbers and density functional
energy gradients within the linear combination of Gaussian-type orbitals
approach. Chem. Phys. Lett..

[43] Murray K. K., Boyd R. K., Eberlin M. N., Langley G. J., Li L., Naito Y. (2013). Definitions of terms
relating to mass spectrometry (IUPAC Recommendations
2013). Pure Appl. Chem..

[44] Bauer C. A., Grimme S. (2016). How to Compute Electron
Ionization Mass Spectra from
First Principles. J. Phys. Chem. A.

[45] Tully J. C. (1990). Molecular
dynamics with electronic transitions. J. Chem.
Phys..

[46] Fabiano E., Keal T., Thiel W. (2008). Implementation
of surface hopping
molecular dynamics using semiempirical methods. Chem. Phys..

[47] Granucci G., Persico M. (2007). Critical appraisal of the fewest switches algorithm
for surface hopping. J. Chem. Phys..

[48] Katritzky A. R., Szafran M., Pfister-Guillouzo G. (1990). The tautomeric equilibria of thio
analogues of nucleic acid bases. Part 3. Ultraviolet photoelectron
spectra of 2-thiouracil and its methyl derivatives. J. Chem. Soc., Perkin Trans. 2.

[49] Khvorostov A., Lapinski L., Rostkowska H., Nowak M. J. (2005). UV-Induced Generation
of Rare Tautomers of 2-Thiouracils: A Matrix Isolation Study. J. Phys. Chem. A.

[50] Rostkowska H., Lapinski L., Nowak M. J. (2024). Intramolecular hydrogen-atom tunneling
in matrix-isolated heterocyclic compounds: 2-thiouracil and its analogues. Phys. Chem. Chem. Phys..

[51] Castrovilli M. C., Trabattoni A., Bolognesi P., O’Keeffe P., Avaldi L., Nisoli M., Calegari F., Cireasa R. (2018). Ultrafast
Hydrogen Migration in Photoionized Glycine. J. Phys. Chem. Lett..

[52] Giuliano B. M., Feyer V., Prince K. C., Coreno M., Evangelisti L., Melandri S., Caminati W. (2010). Tautomerism in 4-Hydroxypyrimidine,
S-Methyl-2-thiouracil, and 2-Thiouracil. J.
Phys. Chem. A.

[53] NIST Mass Spectrometry Data Center. William, E. W. , director. “Mass Spectra” in NIST Chemistry WebBook, NIST Standard Reference Database Number 69; Linstrom, P. J. ; Mallard, W. G , Eds.; National Institute of Standards and Technology: Gaithersburg MD, 20899, 2025. 10.18434/T4D303 (retrieved March 25, 2025).

[54] Benson S. W. (1965). III - Bond
energies. J. Chem. Educ..

[55] Jochim B., DeJesus L., Dantus M. (2022). Ultrafast disruptive probing: Simultaneously
keeping track of tens of reaction pathways. Rev. Sci. Instrum..

[56] Zinchenko K. S., Ardana-Lamas F., Seidu I., Neville S. P., van der
Veen J., Lanfaloni V. U., Schuurman M. S., Wörner H. J. (2021). Sub-7-femtosecond
conical-intersection dynamics probed at the carbon K-edge. Science.

[57] Roy B., Titov E., Saalfrank P. (2024). Nonadiabatic Photodynamics of Amantadine
and 1-Cyanoadamantane Cations. ChemPhysChem.

[58] Macis D., Granucci G., Persico M. (2025). The time-resolved
photoelectron spectrum
of trans-azobenzene and its relationship with the photoisomerization
mechanism: A surface hopping simulation with determination of Dyson
orbitals. J. Chem. Phys..

[59] Mayer, D. ; Titov, E. ; Lever, F. ; Mehner, L. ; Murillo-Sánchez, M. L. ; Walz, C. ; Bozek, J. ; Saalfrank, P. ; Gühr, M. Valence photoelectron spectra of thiouracils in the gas phase. J. Chem. Phys. 2025, accepted (arXiv:2505.07361) 10.48550/arXiv.2505.07361. 40815564

[60] Wigner E. (1932). On the Quantum
Correction For Thermodynamic Equilibrium. Phys.
Rev..

