# Supplementary material for: Dissociative Photoionization of 2‑Thiouracil and 4‑Thiouracil: A Molecular Dynamics Study
Source: J Phys Chem A. 2025 Jul 31;129(32):7352–64. doi: 10.1021/acs.jpca.5c03342 (PMC12359108; doi:10.1021/acs.jpca.5c03342)
Supplement: Supplementary file 1 [file jp5c03342_si_001.pdf]

Supporting Information for
Dissociative Photoionization of 2-Thiouracil and 4-Thiouracil: A Molecular Dynamics Study

Bonasree Roy[1], Evgenii Titov[1], Matthew S. Robinson[2], Markus Gühr[3,4], Peter Saalfrank[1]

[1] *University of Potsdam, Institute of Chemistry, Karl-Liebknecht-Straße 24–25, 14476 Potsdam, Germany*

[2] *European XFEL, Holzkoppel 4, 22869 Schenefeld, Germany*

[3] *Deutsches Elektronen-Synchrotron (DESY), Notkestraße 85, 22607 Hamburg, Germany*

[4] *Institut für Physikalische Chemie, Universität Hamburg, Grindelallee 117, 20146 Hamburg, Germany*

July 14, 2025

Contents

S1 Photodissociative fragments of 2-TU$^+$ **S2**

S2 Tautomers of 2-TU **S7**

S2.1 Tautomer IV S8

S2.2 Tautomer energetics S9

S3 Photodissociative fragments of 4-TU$^+$ **S12**

S4 Relative abundances of cations at different photon energies for 2-TU$^+$ and 4-TU$^+$ **S17**

S5 Optimized geometries of 2-TU and 4-TU **S19**

S6 SH simulations for 2-TU$^+$ **S21**

S1 Photodissociative fragments of 2-TU$^+$

Figure S1: Structures of photodissociative fragments (neutral and cationic) of 2-TU$^+$ obtained from MD simulations at the OM2 level over a 10 ps time frame at different energies. The different groups refer to different fragmentation groups. Their masses sum up to 128 amu.

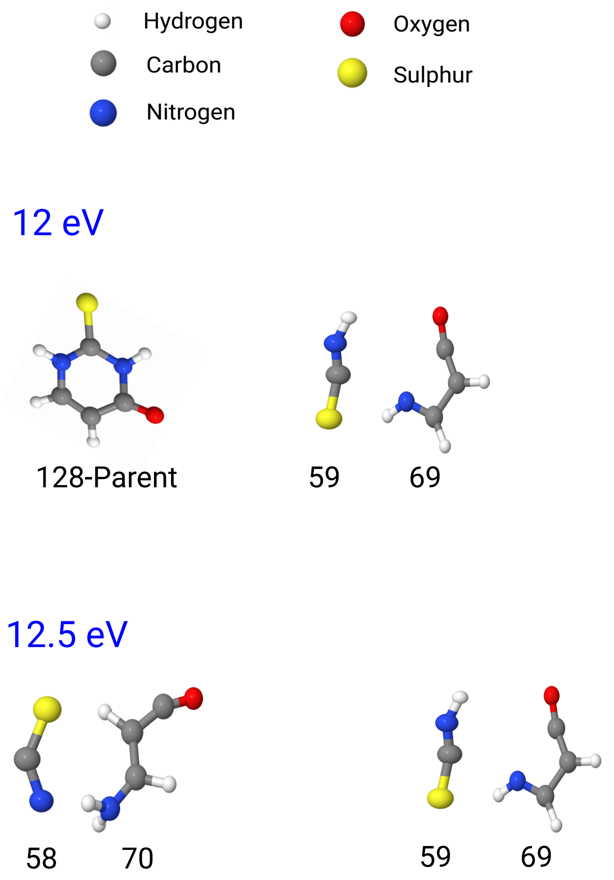

Continuation of Figure S1

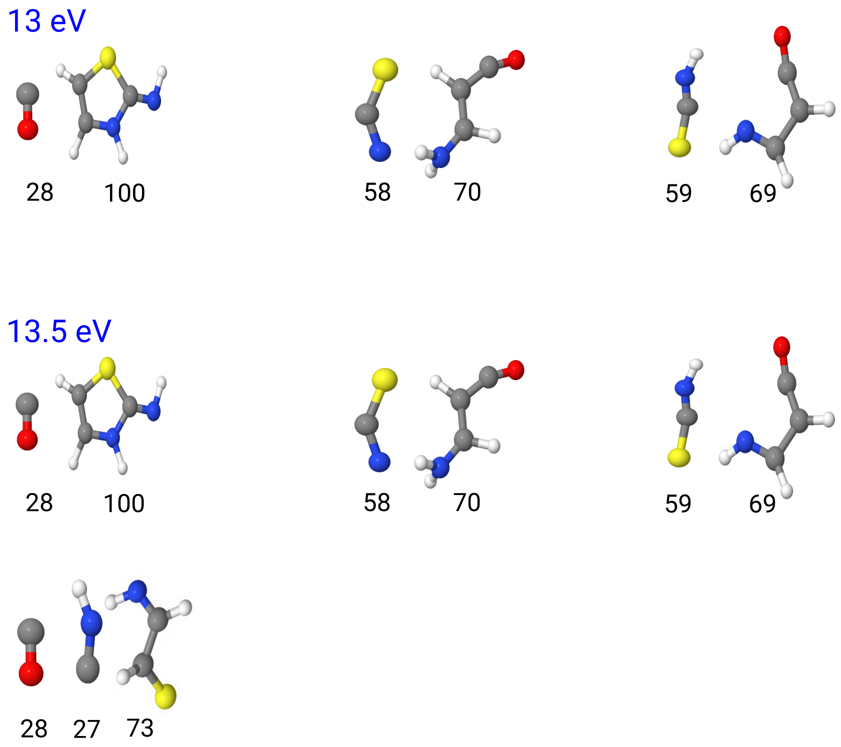

Continuation of Figure S1

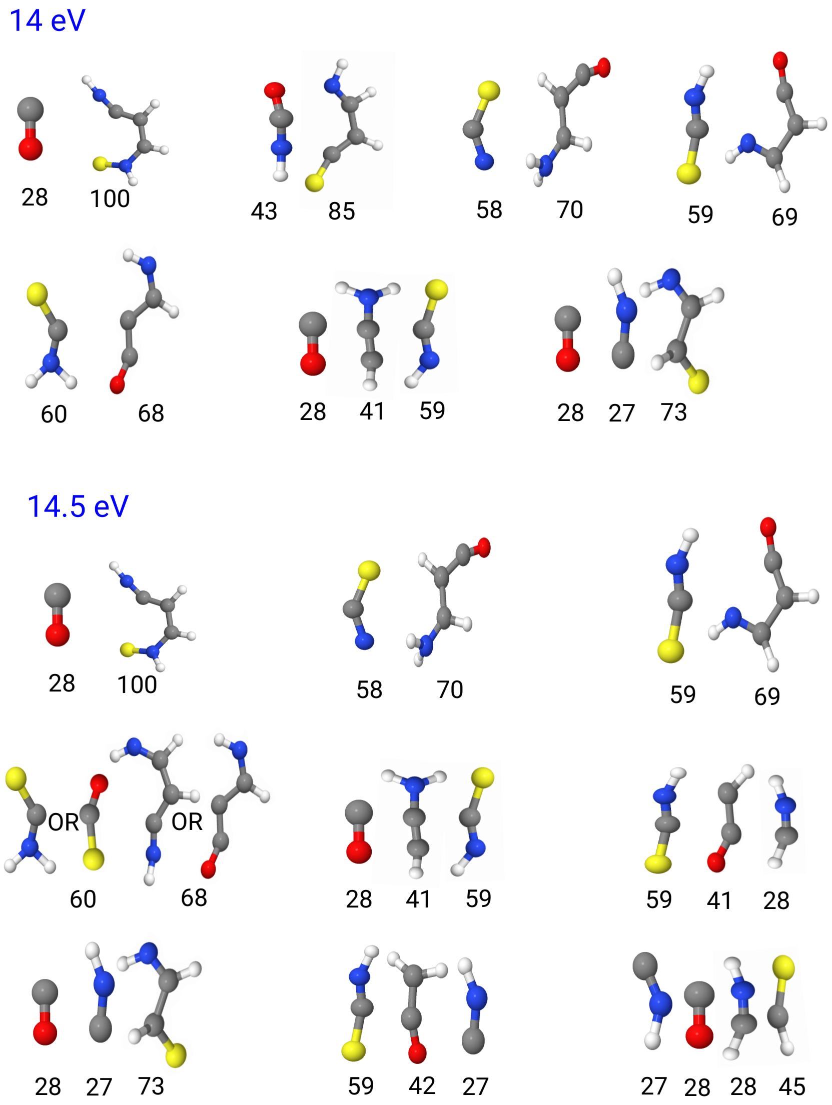

Continuation of Figure S1

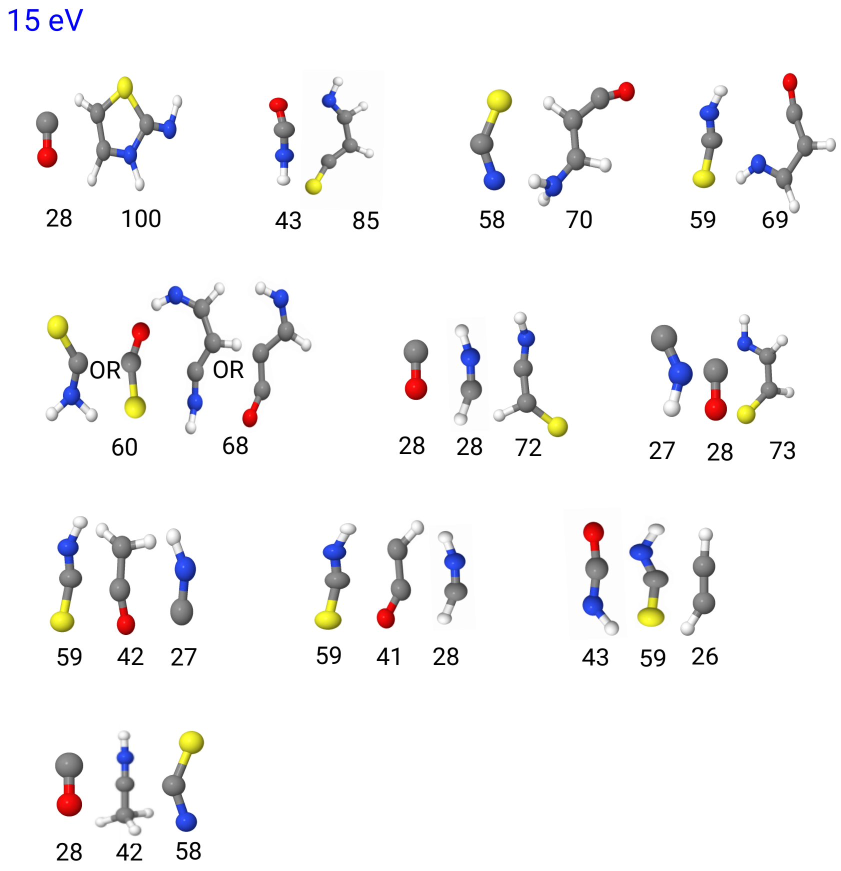

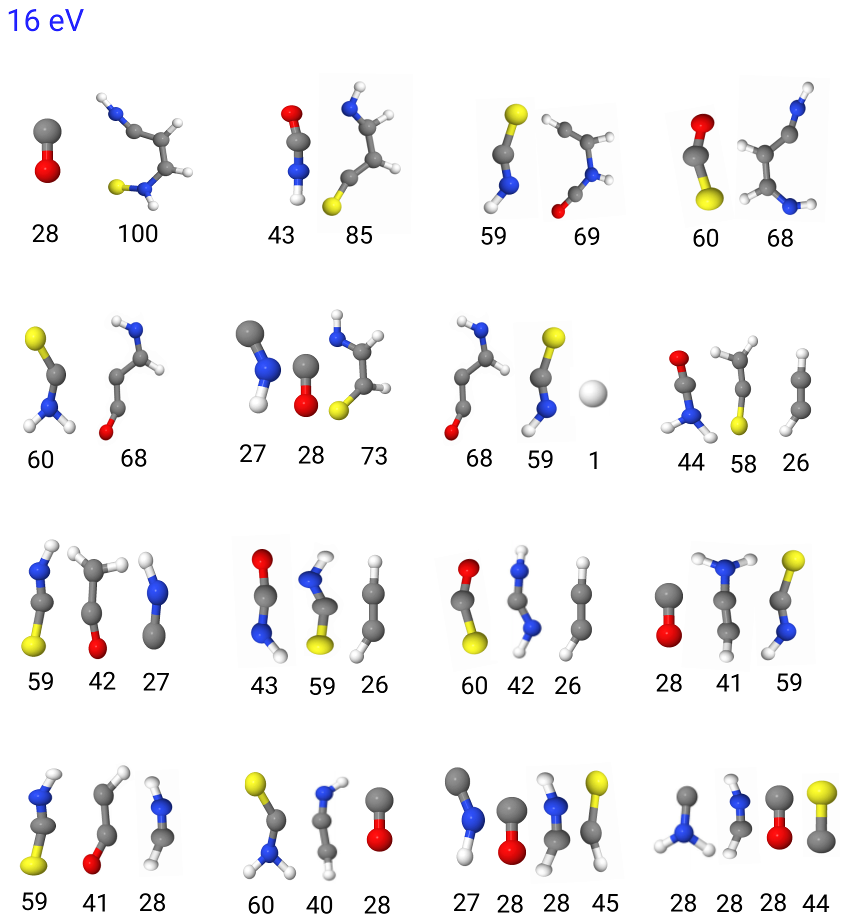
16 eV
28 100
43 85
59 69
60 68
60 68
27 28 73
68 59 1
44 58 26
59 42 27
43 59 26
60 42 26
28 41 59
59 41 28
60 40 28
27 28 28 45
28 28 28 44

S2 Tautomers of 2-TU

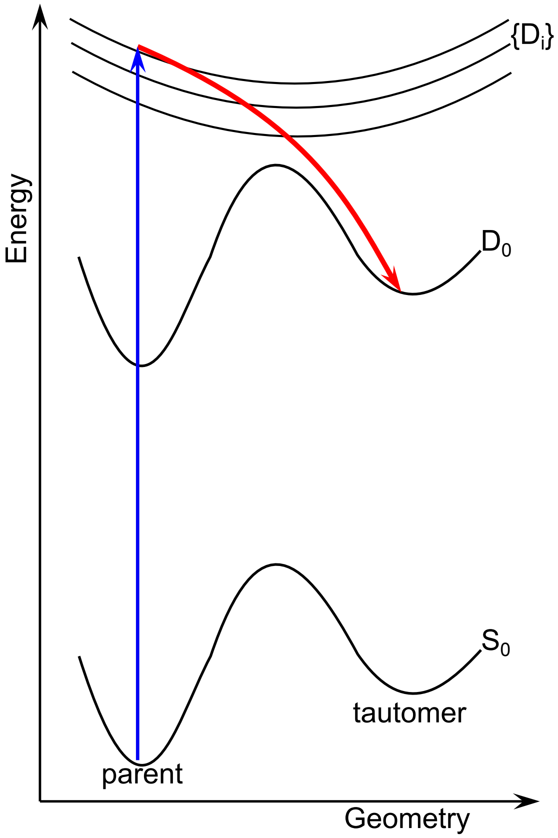

Figure S2: Hypothesis used in this work: Photoionization-induced proton transfer leads to formation of a tautomer. This tautomer then fragments yielding the 95 amu fragment (among others).

S2.1 Tautomer IV

The introduction of UV energy may result in the formation of tautomers. Tautomers II and III exhibited a higher yield of fragments with a m/z ratio of 95 amu, whereas tautomer IV (Fig. S3) produced only a minimal amount of the 95 amu fragment, and this occurred only at an elevated energy of 16 eV, as shown in the mass spectrum in Fig. S4.

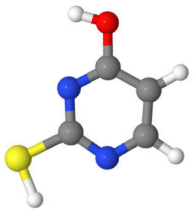

Figure S3: Tautomer IV of 2-Thiouracil

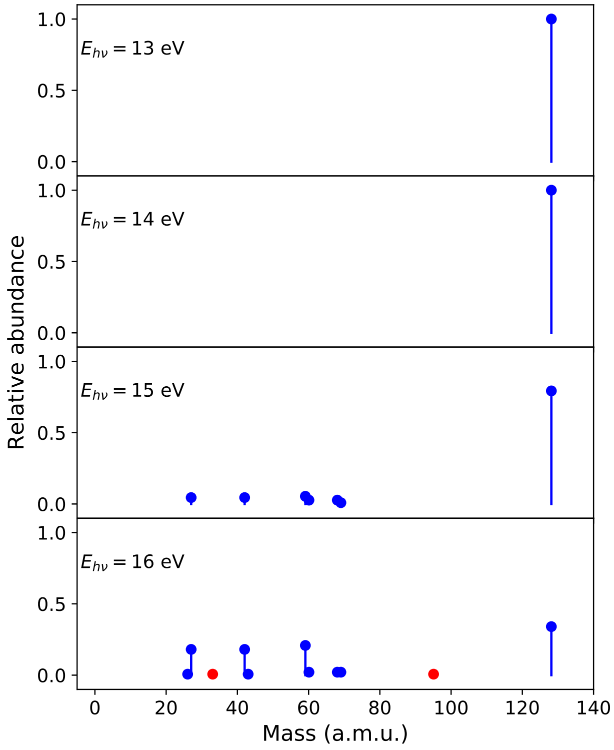

Figure S4: Relative abundance of fragments for various photon energies of tautomer IV of 2-Thiouracil: the red intensities indicate the fragments 33 and 95 amu.

S2.2 Tautomer energetics

In this subsection, we provide details on relative energies of tautomers shown in Fig. S5. The energies in the neutral (S_0) and cationic (D_0) states are presented in Fig. S6. The transition state of the proton transfer reaction I $\leftrightarrow$ IIa and its energetics is shown in Fig. S7.

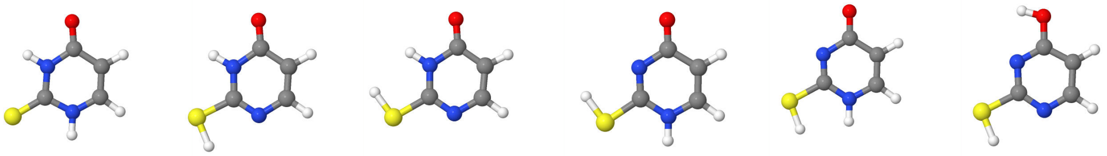

I IIa IIb IIIa IIIb IV

Figure S5: Tautomers of 2-thiouracil considered below.

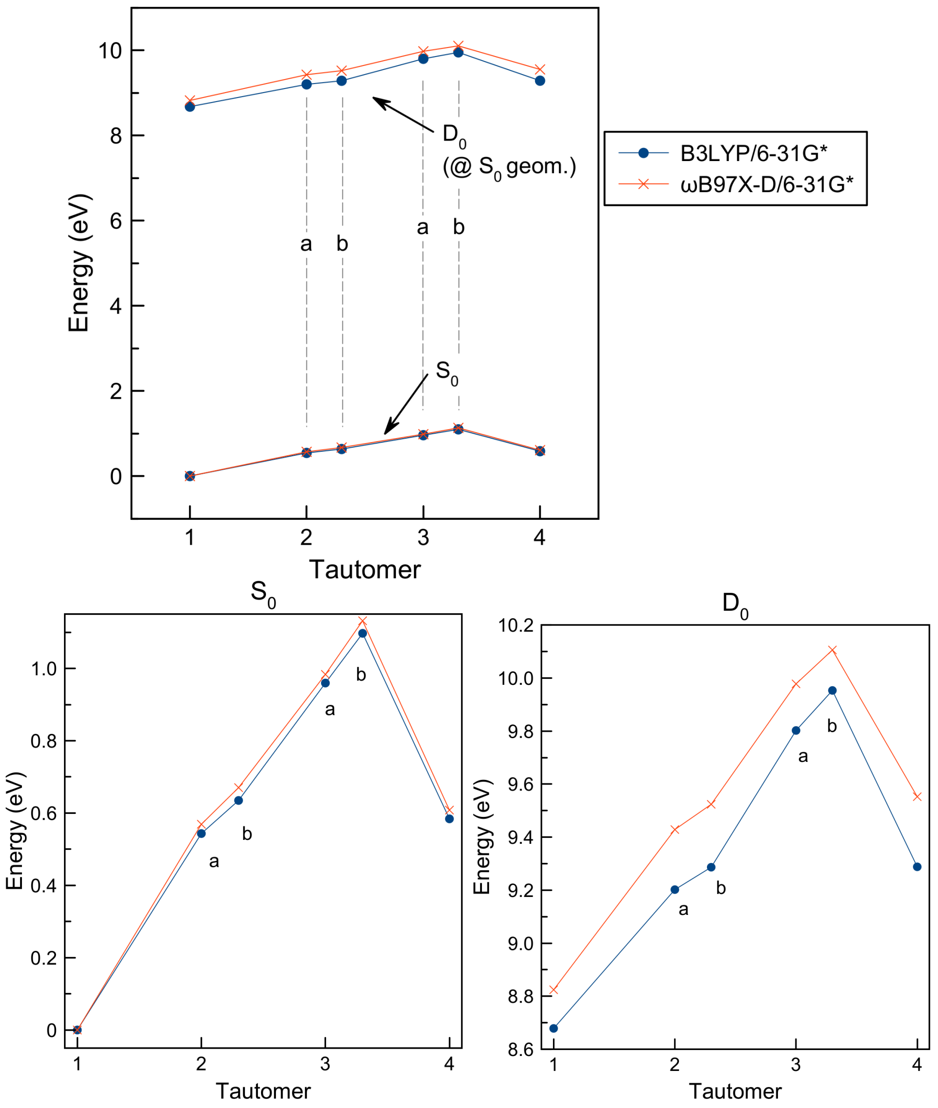

Figure S6: Top: Neutral (S_0) and cation (D_0 @ S_0 geometry) energies of tautomers with respect to the S_0 energy of tautomer I (labeled as 1 in the figure; tautomers are labeled with arabic numerals on "x" axis). Bottom: zoomed in figures for S_0 and D_0.

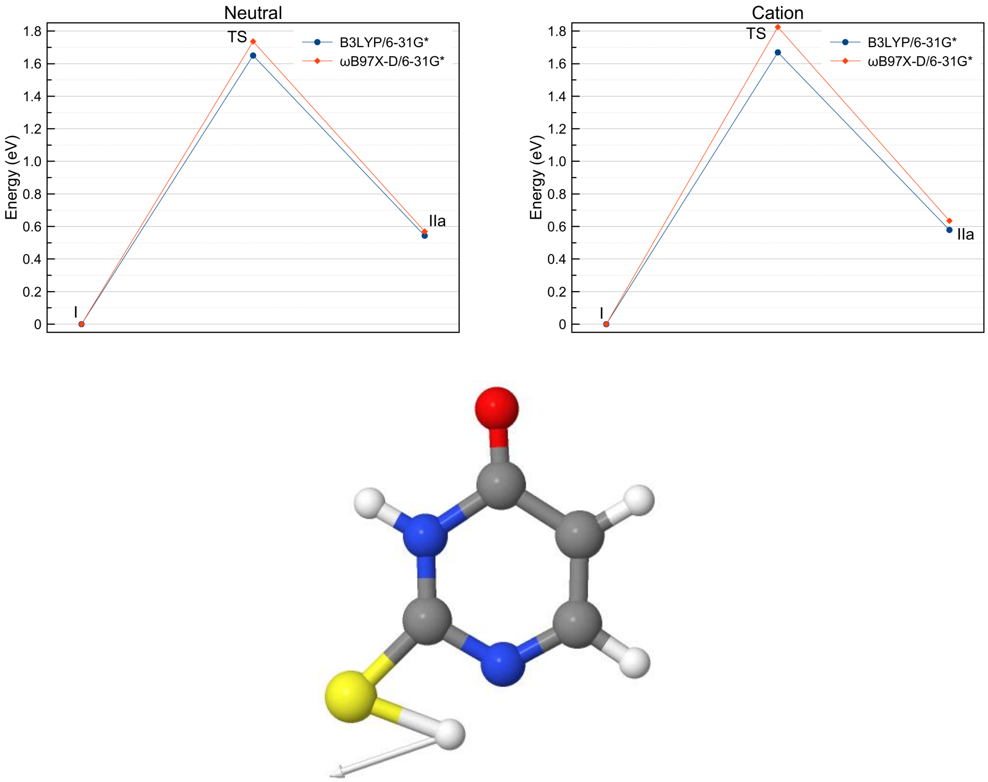

Figure S7: Transition state of the proton transfer reaction I ↔ IIa. Top left: neutral. Top right: cation. SCF energies (PES points) are plotted.

S3 Photodissociative fragments of 4-TU$^+$

Figure S8: Structures of photodissociative fragments (neutral and cationic) of 4-TU$^+$ obtained from MD simulations at the OM2 level over a 10 ps time frame at different energies.

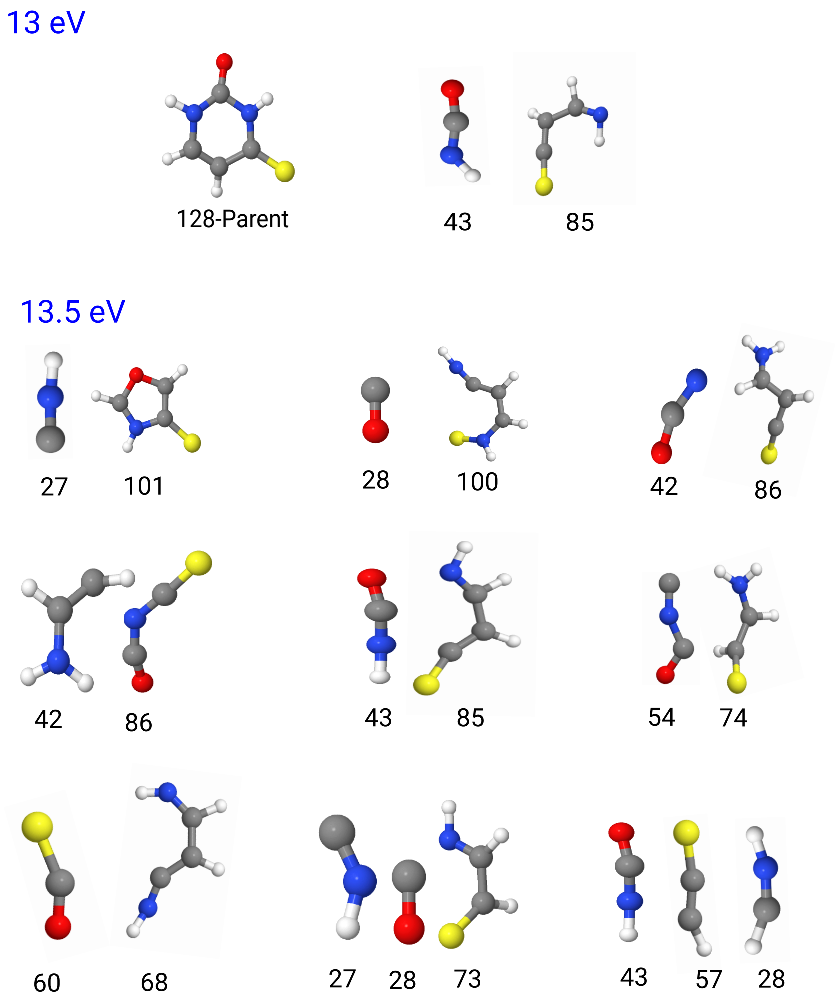

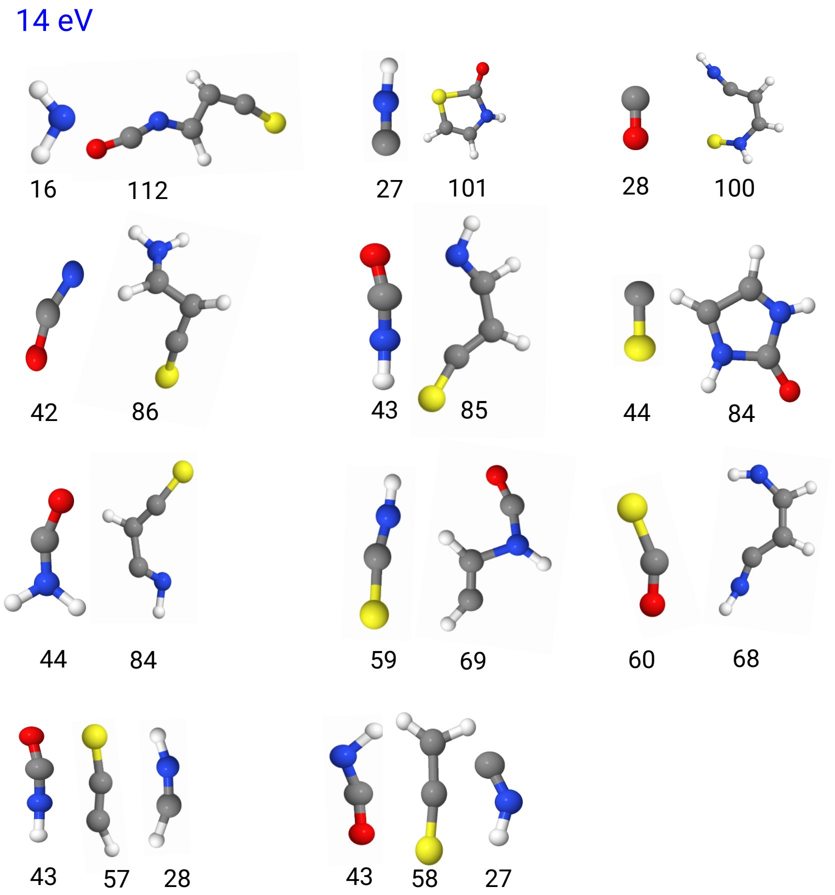
14 eV
16 112
27 101
28 100
42 86
43 85
44 84
44 84
59 69
60 68
43 57 28
43 58 27

Continuation of Figure S8

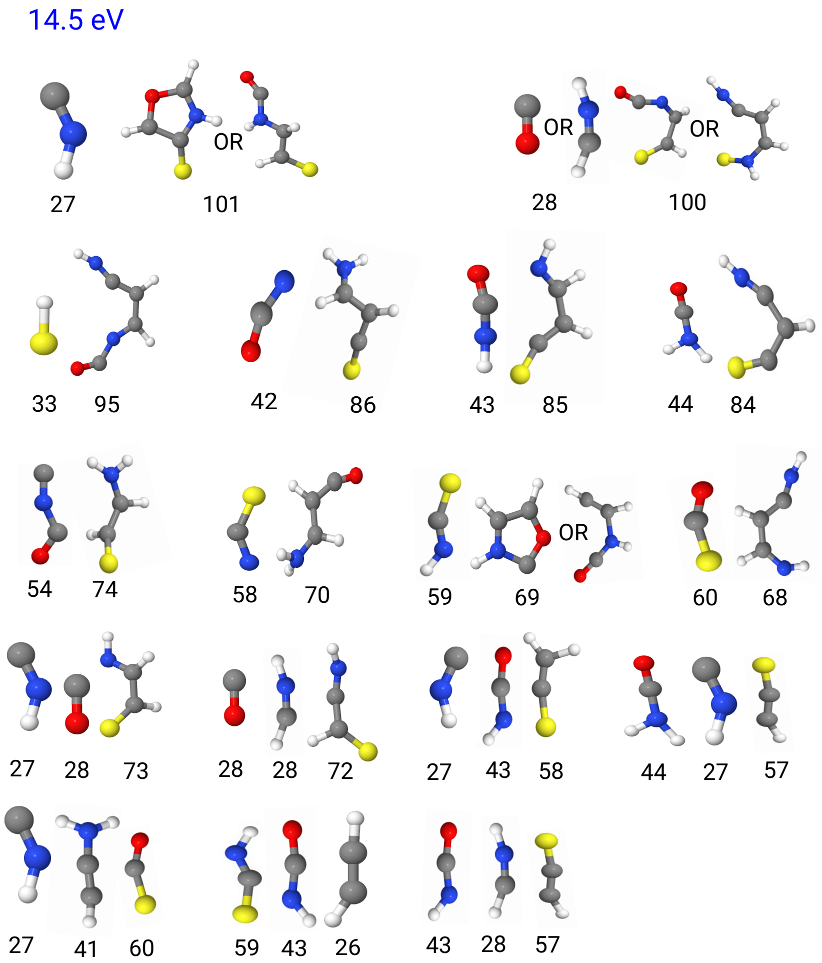

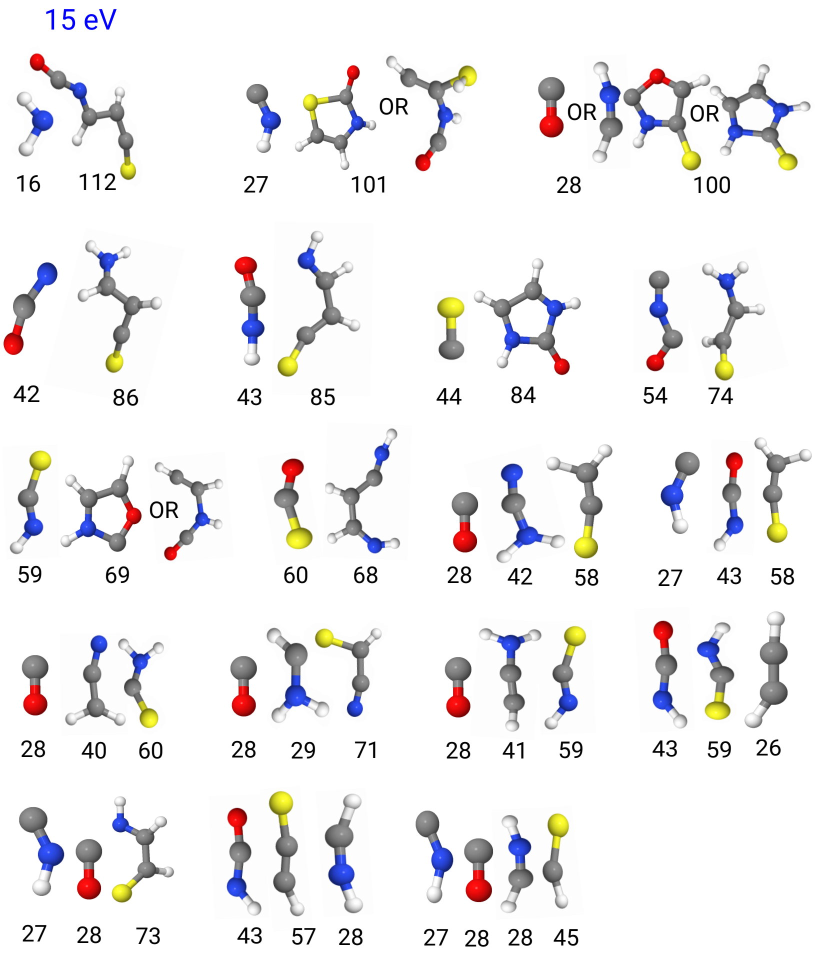
15 eV
16
112
27
101
OR
28
OR
OR
100
42
86
43
85
44
84
54
74
59
69
OR
60
68
28
42
58
27
43
58
28
40
60
28
29
71
28
41
59
43
59
26
27
28
73
43
57
28
27
28
28
45

Continuation of Figure S8

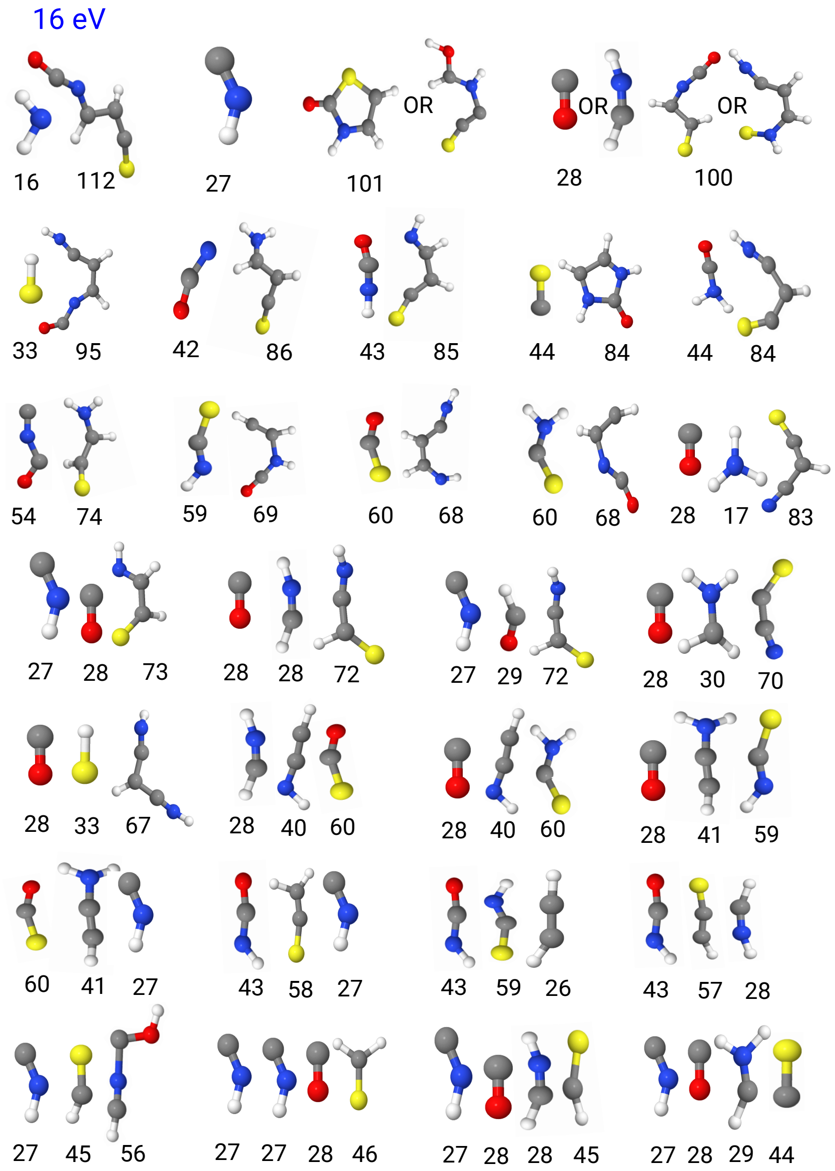

S4 Relative abundances of cations at different photon energies for 2-TU$^+$ and 4-TU$^+$

Table S1: Abundance of cationic fragments for 2-TU and 4-TU, for different photon energies.

Photon energy (eV)	2-TU fragment ions (% Abundance)	4-TU fragment ions (% Abundance)
12	128 amu, parent (99.6)<br>69 amu, C_3NH_3O (0.4)	128 amu, parent (100.0)
12.5	128 amu, parent (96.6)<br>70 amu, C_3NH_4O (0.5)<br>69 amu, C_3NH_3O (2.9)	128 amu, parent (100.0)
13	128 amu, parent (88.2)<br>100 amu, $C_3N_2H_4S$ (0.3)<br>70 amu, C_3NH_4O (1.7)<br>69 amu, C_3NH_3O (9.8)	128 amu, parent (99.1)<br>85 amu, C_3NH_3S (0.9)
13.5	128 amu, parent (66.6)<br>100 amu, $C_3N_2H_4S$ (1.3)<br>73 amu, C_2NH_3S (0.2)<br>70 amu, C_3NH_4O (1.8)<br>69 amu, C_3NH_3O (30.0)	128 amu, parent (94.4)<br>101 amu, C_3H_3NSO (0.2)<br>100 amu, $C_3H_4N_2S$ (0.2)<br>86 amu, C_3H_4NS (0.3)<br>86 amu, C_2NSO (0.1)<br>85 amu, C_3NH_3S (4.3)<br>74 amu, C_2H_4NS (0.1)<br>73 amu, C_2H_3NS (0.1)<br>68 amu, $C_3H_4N_2$ (0.2)<br>28 amu, HCNH (0.1)
14	128 amu, parent (35.8)<br>100 amu, $C_3N_2H_4S$ (1.7)<br>85 amu, C_3NH_3S (0.1)<br>73 amu, C_2NH_3S (0.2)<br>70 amu, C_3NH_4O (2.9)<br>69 amu, C_3NH_3O (59.0)<br>60 amu, $SCNH_2$ (0.2)<br>41 amu, C_2NH_3 (0.1)	128 amu, parent (80.7)<br>112 amu, C_4H_2NSO (0.1)<br>101 amu, C_3H_3NSO (0.1)<br>100 amu, $C_3H_4N_2S$ (1.0)<br>86 amu, C_3H_4NS (0.4)<br>85 amu, C_3NH_3S (16.0)<br>84 amu, C_3H_2NS (0.1)<br>84 amu, $C_3H_4N_2O$ (0.1)<br>69 amu, C_3NH_3O (0.4)<br>68 amu, $C_3H_4N_2$ (0.6)<br>58 amu, C_2H_2S (0.1)<br>28 amu, HCNH (0.4)
14.5	128 amu, parent (11.7)<br>100 amu, $C_3N_2H_4S$ (2.8)<br>73 amu, C_2NH_3S (0.1)<br>70 amu, C_3NH_4O (1.9)<br>69 amu, C_3NH_3O (82.5)<br>68 amu, $C_3H_4N_2$ (0.1)<br>60 amu, $SCNH_2$ (0.6)<br>42 amu, C_2H_2O (0.1)<br>41 amu, C_2NH_3 (0.1)<br>28 amu, HCNH (0.2)	128 amu, parent (56.7)<br>101 amu, C_3H_3NSO (0.2)<br>100 amu, $C_3H_4N_2S$ (1.9)<br>95 amu, $C_4H_3N_2O$ (0.1)<br>86 amu, C_3H_4NS (0.5)<br>85 amu, C_3NH_3S (33.1)<br>84 amu, C_3NH_2S (0.1)<br>74 amu, C_2NH_4S (0.3)<br>73 amu, C_2NH_3S (0.3)<br>72 amu, C_2NH_2S (0.1)<br>70 amu, C_3NH_4O (0.1)<br>69 amu, C_3NH_3O (0.7)<br>68 amu, $C_3H_4N_2$ (1.9)<br>58 amu, C_2H_2S (0.2)<br>57 amu, C_2HS (0.1)<br>43 amu, OCNH (0.2)<br>41 amu, C_2H_3N (0.1)<br>28 amu, HCNH (3.2)

Continuation of Table S1

Photon energy (eV)	2-TU fragment ions (% Abundance)	4-TU fragment ions (% Abundance)
15	128 amu, parent (3.0)	128 amu, parent (32.0)
	100 amu, $C_3N_2H_4S$ (3.1)	112 amu, C_4H_2NSO (0.1)
	85 amu, C_3NH_3S (0.5)	101 amu, C_3H_3NSO (0.2)
	73 amu, C_2NH_3S (0.9)	100 amu, $C_3H_4N_2S$ (1.9)
	72 amu, C_2NH_2S (0.2)	86 amu, C_3H_4NS (1.1)
	70 amu, C_3NH_4O (0.9)	85 amu, C_3NH_3S (43.4)
	69 amu, C_3NH_3O (88.8)	84 amu, $C_3N_2H_4O$ (0.1)
	68 amu, $C_3H_4N_2$ (0.4)	74 amu, C_2NH_4S (0.5)
	60 amu, $SCNH_2$ (0.8)	73 amu, C_2NH_3S (1.3)
	59 amu, SCNH (0.1)	69 amu, C_3NH_3O (1.3)
	58 amu, SCN (0.1)	68 amu, $C_3H_4N_2$ (3.2)
	42 amu, C_2H_2O (0.8)	60 amu, CNH_2S (0.1)
	28 amu, HCNH (0.4)	59 amu, CNHS (0.5)
		58 amu, C_2H_2S (1.6)
		41 amu, C_2H_3N (0.1)
		29 amu, CH_3N (0.1)
		28 amu, HCNH (12.6)
16	128 amu, parent (0.5)	128 amu, parent (2.5)
	100 amu, $C_3N_2H_4S$ (2.0)	112 amu, C_4H_2NSO (0.1)
	85 amu, C_3NH_3S (0.3)	101 amu, C_3H_3NSO (0.2)
	73 amu, C_2NH_3S (1.2)	100 amu, $C_3H_4N_2S$ (3.9)
	69 amu, C_3NH_3O (80.7)	95 amu, $C_4H_3N_2O$ (0.4)
	68 amu, $C_3H_4N_2$ (0.3)	86 amu, C_3H_4NS (0.9)
	68 amu, C_3NH_2O (0.9)	85 amu, C_3NH_3S (27.2)
	60 amu, $SCNH_2$ (1.7)	84 amu, C_3NH_2S (0.2)
	59 amu, SCNH (2.7)	84 amu, $C_3N_2H_4O$ (0.1)
	44 amu, CNH_2O (0.2)	83 amu, C_3NHS (0.2)
	42 amu, CN_2H_2 (0.2)	74 amu, C_2NH_4S (0.6)
	42 amu, C_2H_2O (4.8)	73 amu, C_2NH_3S (2.3)
	41 amu, C_2NH_3 (0.5)	72 amu, C_2NH_2S (0.8)
	40 amu, C_2NH_2 (0.2)	69 amu, C_3NH_3O (0.8)
	28 amu, HCNH (3.6)	68 amu, $C_3H_4N_2$ (1.3)
		67 amu, $C_3H_3N_2$ (0.5)
		60 amu, CNH_2S (0.7)
		59 amu, CNHS (3.6)
		58 amu, C_2H_2S (6.2)
		57 amu, C_2HS (0.2)
		56 amu, C_2H_2NO (0.1)
		46 amu, CH_2S (0.1)
		41 amu, C_2H_3N (0.6)
		30 amu, CH_4N (0.1)
		29 amu, CH_3N (0.1)
		28 amu, HCNH (46.1)

S5 Optimized geometries of 2-TU and 4-TU

Table S2: Optimized geometries of 2-TU at the semiemperical OM2 method in comparison with optimized geometry at the B3LYP/6-31G* level of theory for selected bond lengths (in Å) and bond angles (in degrees).

2-TU	OM2	B3LYP
N1-C2	1.392	1.379
C2-N3	1.377	1.369
N3-C4	1.412	1.419
C4-C5	1.462	1.458
C5-C6	1.359	1.351
C6-N1	1.378	1.376
N1-H7	1.018	1.011
C2-S8	1.664	1.666
N3-H9	1.019	1.015
C4-O10	1.237	1.218
C5-H11	1.081	1.081
C6-H12	1.102	1.085
S8-C2-N3	123.8°	124.4°
N3-C4-O10	118.5°	120.0°
C5-C6-N1	120.7°	121.7°
C6-N1-C2	124.3°	123.9°
C2-N3-C4	125.4°	128.3°
C6-C5-H11	123.3°	122.0°
N1-C2-N3	114.6°	113.2°
N3-C4-C5	116.0°	113.2°

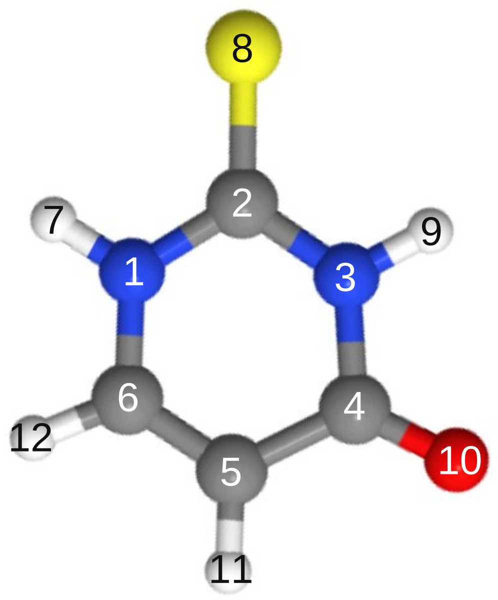

Table S3: Optimized geometries of 4-TU at the semiemperical OM2 method in comparison with optimized geometry at the B3LYP/6-31G* level of theory for selected bond lengths (in Å) and bond angles (in degrees).

4-TU	OM2	B3LYP
N1-C2	1.398	1.394
C2-N3	1.395	1.390
N3-C4	1.387	1.390
C4-C5	1.459	1.443
C5-C6	1.364	1.355
C6-N1	1.373	1.374
N1-H7	1.015	1.011
C2-O8	1.244	1.215
N3-H9	1.019	1.015
C4-S10	1.652	1.663
C5-H11	1.084	1.081
C6-H12	1.101	1.084
O8-C2-N3	122.6°	124.0°
N3-C4-S10	121.4°	120.9°
C5-C6-N1	120.4°	121.4°
C6-N1-C2	122.6°	123.4°
C2-N3-C4	125.1°	128.2°
C6-C5-H11	121.5°	121.3°
N1-C2-N3	115.9°	112.8°
N3-C4-C5	115.2°	114.1°

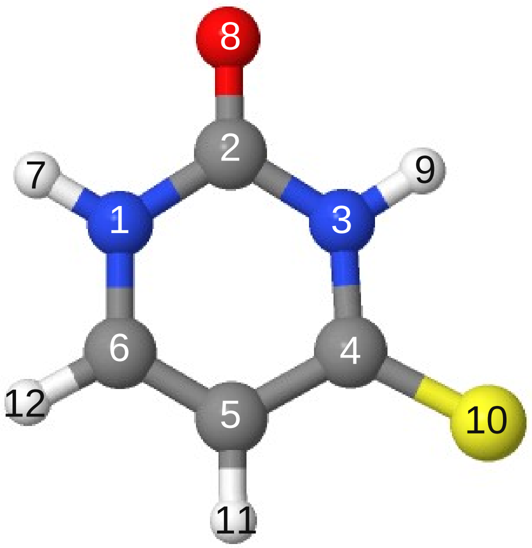

S6 SH simulations for 2-TU$^+$

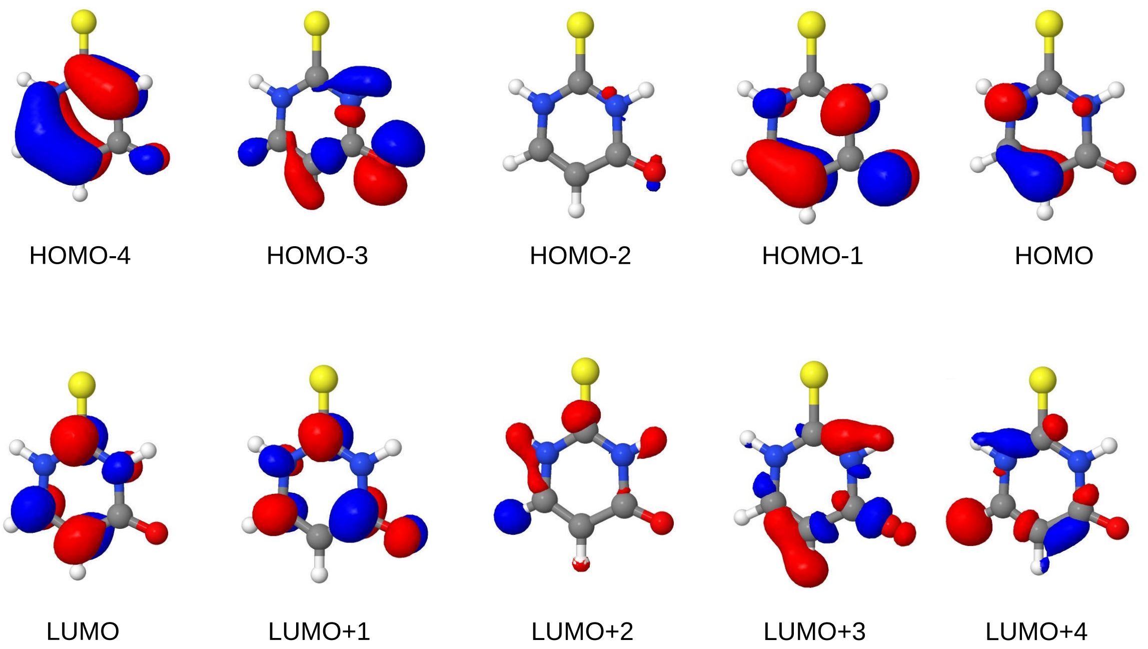

Figure S9: Orbitals considered for active space (5×5) in the SH simulations.

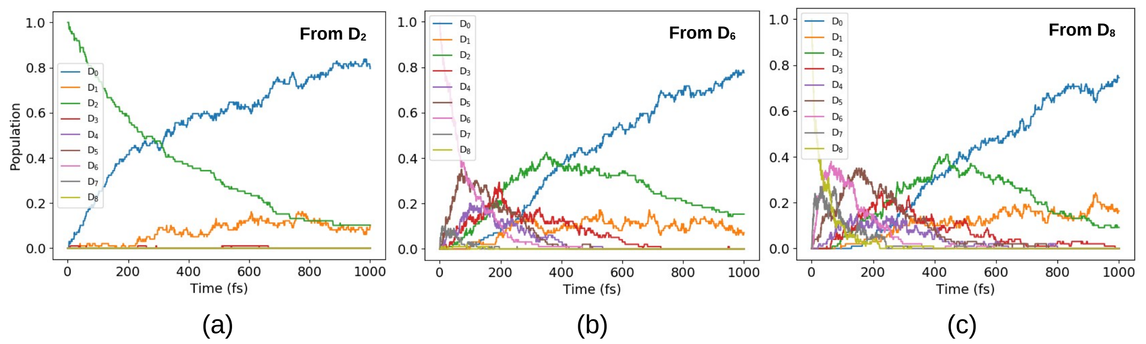

Figure S10: Adiabatic electronic state populations (at the ROOM2/CISD(5×5) level) as a function of time for the three cases studied, for 2-TU$^+$. (a) SH simulations starting from the D_2 state, (b) SH simulations starting from the D_6 state, (c) SH simulations starting from the D_8 state.
